# The mechanisms of specialized pro-resolving mediators in pain relief: neuro-immune and neuroglial regulations

**DOI:** 10.3389/fimmu.2025.1634724

**Published:** 2025-10-30

**Authors:** Yushan Chen, Xuewei Wu, Jiaqi Li, Yuxuan Ren, He Miao, Xiaojie Zhai, Changshun Huang, Xiaowei Chen

**Affiliations:** ^1^ Department of Anesthesiology, The First Affiliated Hospital of Ningbo University, Ningbo, China; ^2^ Health Science Center, Ningbo University, Ningbo, China

**Keywords:** SPM, pain, resolvin, neuro-immune interactions, resolution of inflammation

## Abstract

Chronic pain remains a significant global health challenge. Current anti-nociceptive therapies often fail to provide adequate relief and are associated with adverse side effects, underscoring the need for novel therapeutic approaches. Specialized pro-resolving mediators (SPMs)—bioactive lipid compounds derived from omega-3 and omega-6 fatty acids—have recently garnered attention as potential agents for pain management due to their dual anti-inflammatory and inflammation-resolving properties. This review explores the multifaceted anti-nociceptive effects of SPMs, focusing on their mechanisms of action in diverse pain models, including neuropathic, inflammatory, cancer-induced, postoperative, and spontaneous pain. We highlight the distinct roles of specific SPMs, such as Resolvin D1 (RvD1), Resolvin E1 (RvE1), and Maresin 1 (MaR1), in modulating pain pathways through mechanisms such as suppression of inflammatory cytokines, modulation of transient receptor potential (TRP) channels, and interactions with immune cells to resolve inflammation. Additionally, we discuss the implications of sexual dimorphism in SPM efficacy, endogenous SPM biosynthesis, and therapeutic strategies involving omega-3 fatty acid supplementation. While preclinical studies demonstrate the therapeutic promise of SPMs, critical gaps persist in understanding their precise mechanisms, long-term safety, and translational potential. This review emphasizes the need for rigorous preclinical and clinical research to elucidate SPMs’ role in managing recalcitrant pain conditions, with the aim of advancing targeted, non-opioid pain therapies.

## Graphical Abstract

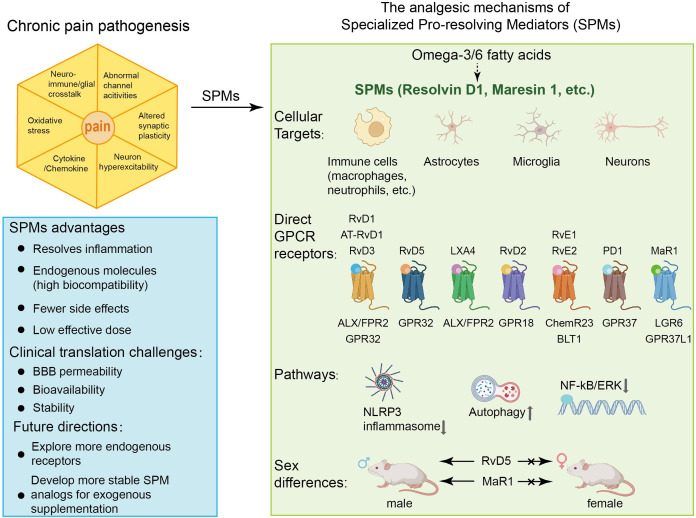

## 1 Introduction

Pain is defined as “an unpleasant sensory and emotional experience associated with, or resembling that associated with, actual or potential tissue damage” (1). Chronic pain, in particular, represents a major global health challenge, severely impacting quality of life and imposing substantial socioeconomic burdens (2). For instance, A meta-analysis estimated that the prevalence of chronic pain in the general population of developing countries is 18%, while in Germany it is 18.4%, 21.5% in Hong Kong, 24.4% in Norway, 19% in Denmark, and 19% and 20.4% in the United States (3). The economic burden of chronic pain is equally staggering. In the United States, approximately one-third of the population is affected, with annual costs estimated at US$560–635 billion (4). In Australia, individuals with chronic pain incur average annual costs of AU$22,588–42,979 per person when non-financial costs are considered (4). However, managing this pervasive condition remains profoundly challenging. First-line treatments, including antidepressants (e.g., duloxetine, amitriptyline) and anticonvulsants (e.g., gabapentin, pregabalin) for neuropathic pain, and non-steroidal anti-inflammatory drugs (NSAIDs)/cyclooxygenase-2 (COX-2) inhibitors for inflammatory pain, often yield incomplete relief and are plagued by significant side effects, ranging from sedation and gastrointestinal toxicity to the risks of dependence on opioids. These limitations of conventional analgesics underscore the urgent, unmet need for novel, mechanism-driven therapies that not only effectively suppress pain but also promote its active resolution with improved safety profiles.

Specialized pro-resolving mediators (SPMs) are endogenous bioactive compounds biosynthesized from omega-3 [e.g., eicosapentaenoic (EPA), docosahexaenoic (DHA)] and omega-6 (e.g., arachidonic acid) polyunsaturated fatty acids. This family includes resolvins, protectins, maresins, and lipoxins (5). Unlike conventional anti-inflammatory agents, SPMs do not suppress immune function; instead, they orchestrate inflammatory resolution by actively promoting homeostasis through receptor-mediated pathways (6). Their multifaceted roles include resolving inflammation (7, 8), facilitating tissue and organ repair (9), modifying lipidome (8), providing anti-infection properties (10, 11), alleviating depressive symptoms (12), modulating tumor progression (13), preventing atherosclerosis (14), and enhancing the healing response following vascular injury (15). To date, 43 distinct SPMs have been identified. Though the quantity is relatively small, their potent pro-resolving and anti-inflammatory actions form the basis of resolution pharmacology—a therapeutic strategy focused on restoring physiological balance rather than broadly inhibiting inflammation (16). SPMs are widely distributed throughout the human body. They have been detected in multiple tissues, including the brain, adipose tissue, placenta, lymph nodes, and spleen. Notably, the brain exhibits the highest recorded tissue concentrations (380–1,800 pg per mg protein). SPMs are also present in various biological fluids—such as exhaled breath condensate, synovial fluid, serum, plasma, urine, cerebrospinal fluid, breast milk, and sputum (particularly in the context of cystic fibrosis). Among these fluids, breast milk (10–27,000 pM), serum (14–6,000 pM), and exhaled breath condensate (6,000 pM) contain the highest reported concentrations (17). Notably, their concentrations in serum rise during acute inflammatory responses, suggesting a dynamic role in resolving inflammation (18).

Emerging evidence suggests that SPMs exert anti-nociceptive effects within the nervous system by targeting specific receptors. Direct receptors currently identified include: lipoxin A receptor/formyl-peptide receptor 2 (ALX/FPR2), activated by RvD1, RvD3, AT-RvD1, and lipoxins (19–21); Chemerin receptor 23 (ChemR23) and Leukotriene B4 receptor 1, both modulated by RvE1 and RvE2 (22, 23); Resolvin D1 Receptor 1/G-protein-coupled receptor 32 (DRV1/GPR32), engaged by RvD1, RvD3, RvD5, and AT-RvD1 (24–26); GPR18 influenced by RvD2 (27); MaR1 acts on G protein-coupled receptor 37 like 1 (GPR37L1) and LGR6 as its specific receptor (26, 28); PD1 enhances macrophage phagocytosis through GPR37 (29). Beyond the nervous system, SPMs also demonstrate therapeutic potential in modulating cancer progression, suppressing inflammation, accelerating tissue repair and regeneration, and providing antioxidant protection through interactions with GPR101, GPR120, RORα, LGR6, CysLT1, and AhR (30–34) (See **Table 1** and graphic abstract).

**Table 1:** SPM and its structure, receptors, and whether it has anti-nociceptive effects.

Resovins	SPM molecules	Structure	Endogenous receptors	Pain related
	Resolvin D1(RvD1)	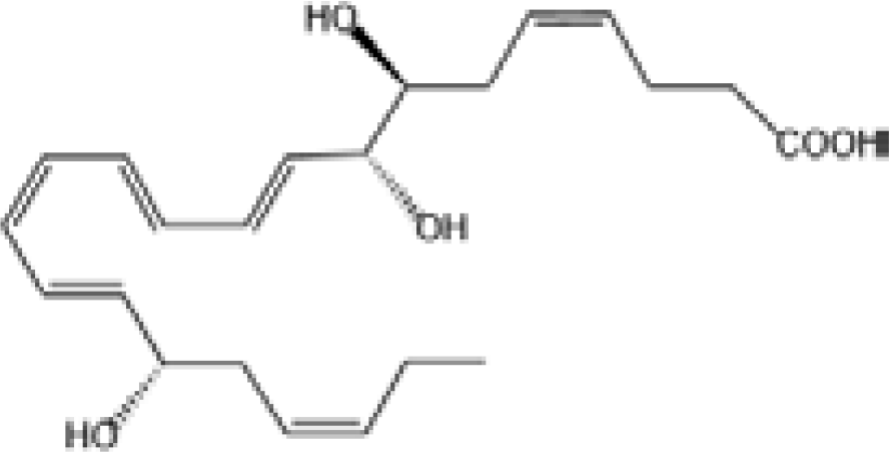	DRV1/GPR32 and ALX/FPR2 (133, 134)	YES (113)
	Resolvin D2(RvD2)	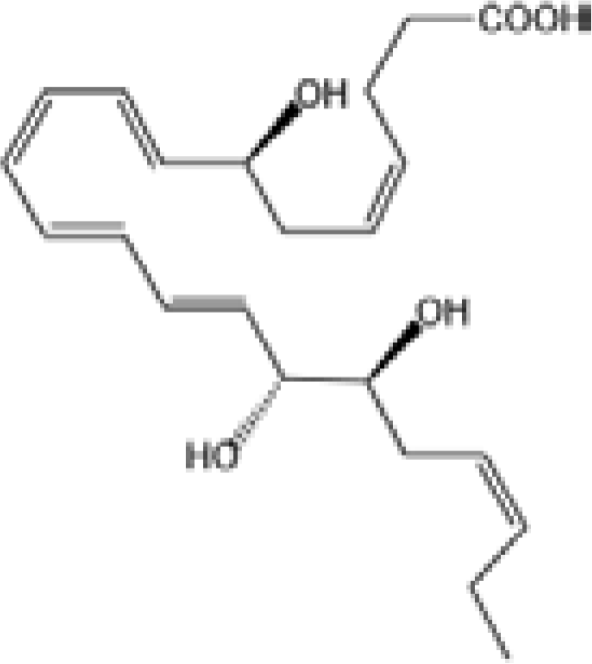	DRV2/GPR18 (134)	YES (106)
	Resolvin D3(RvD3)	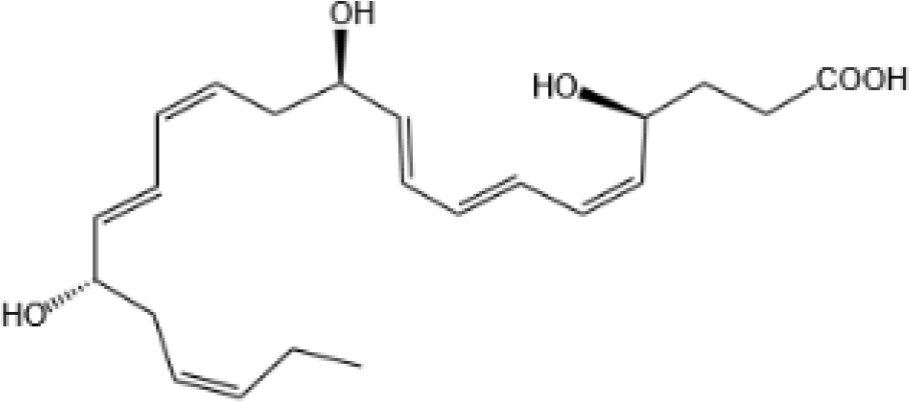	DRV1/GPR32 and ALX/FPR2 (134)	YES (21)
	Resolvin D4(RvD4)	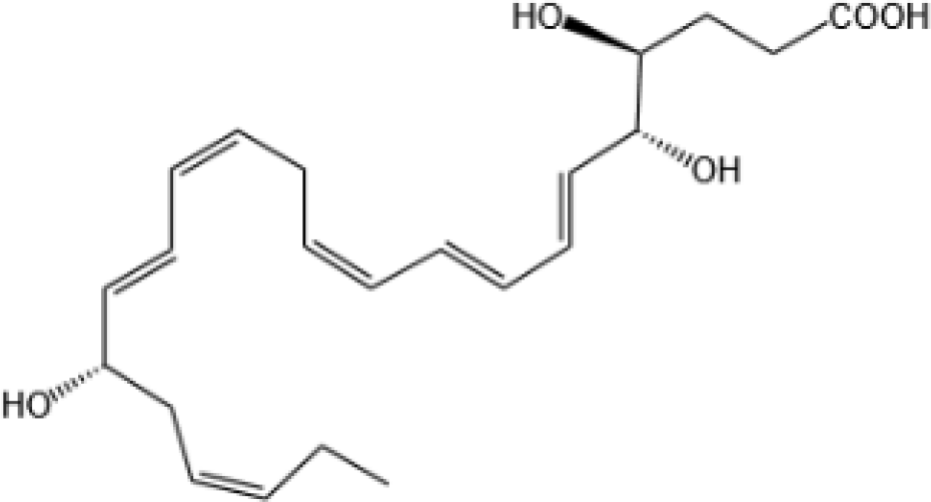	N/A	NO (25)
	Resolvin D5(RvD5)	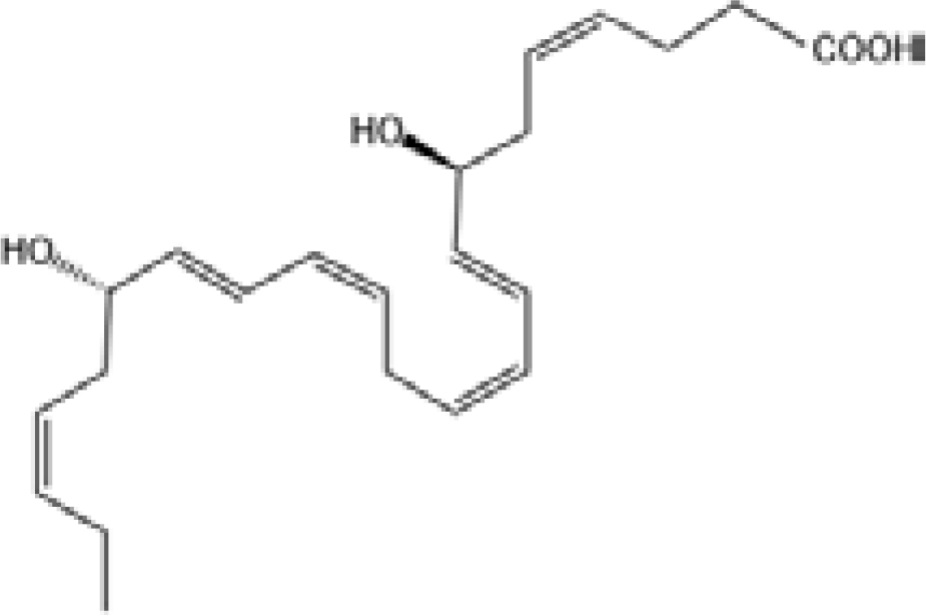	DRV1/GPR32 (134)	YES (25)
	Resolvin D6(RvD6)	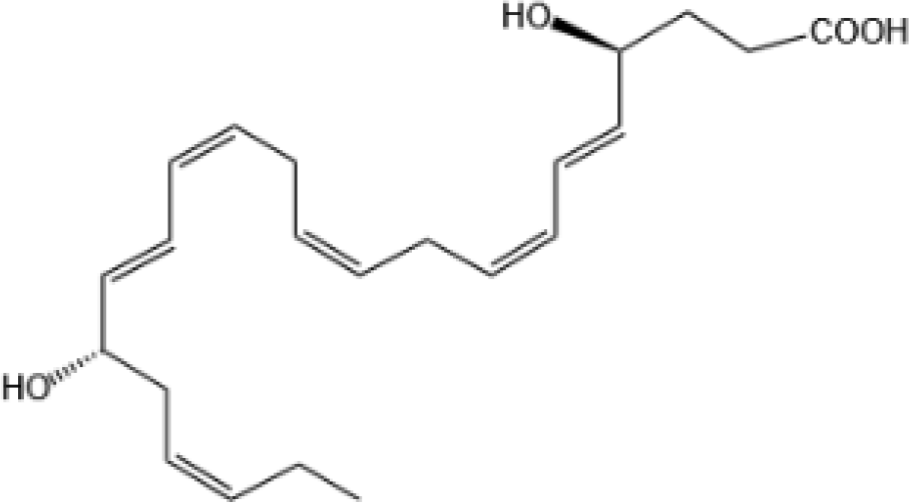	N/A	N/A
	Resolvin T1 (RvT1)	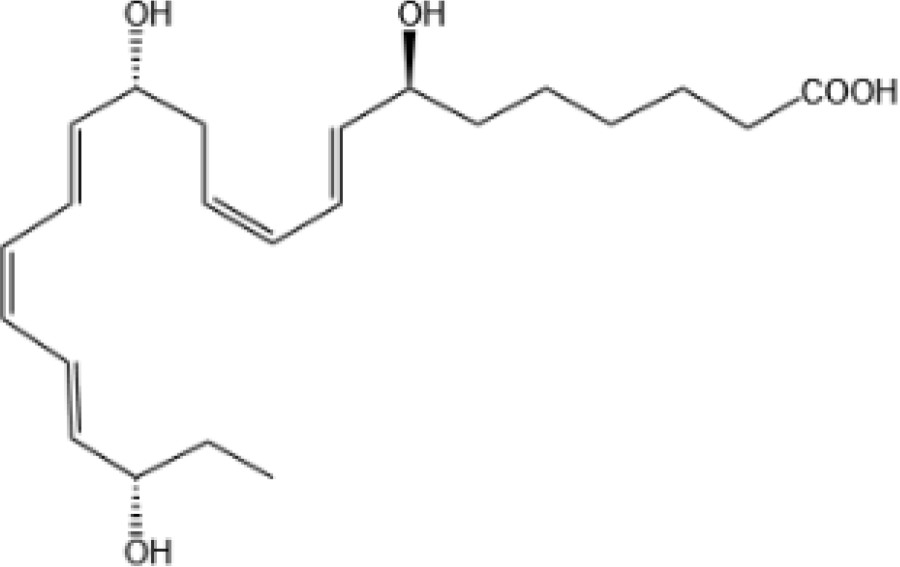	N/A	N/A
	Resolvin T2 (RvT2)	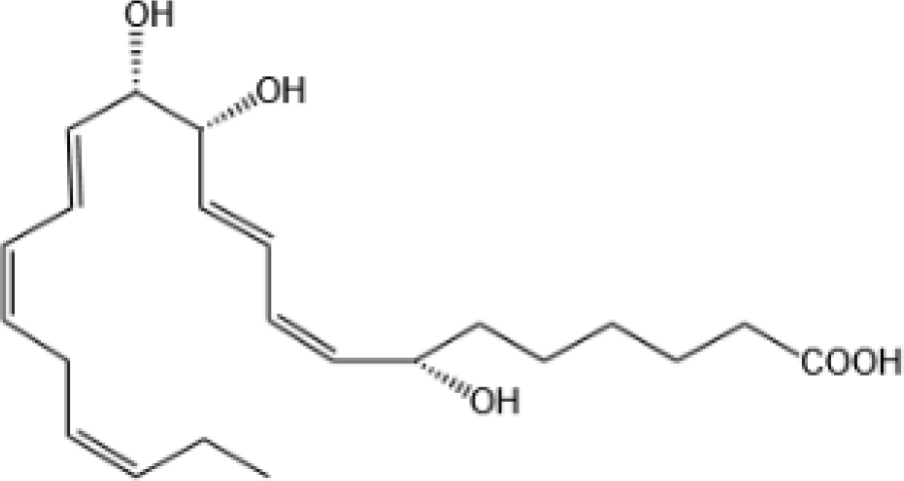	N/A	N/A
	Resolvin T3 (RvT3)	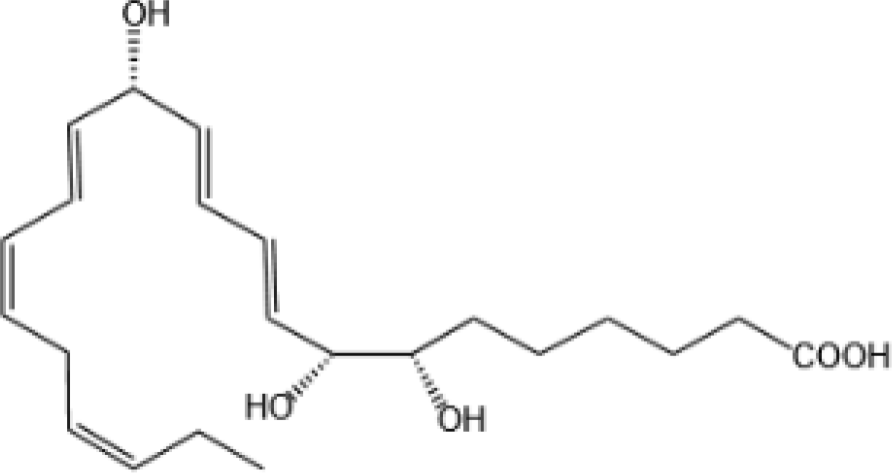	N/A	N/A
	Resolvin T4 (RvT4)	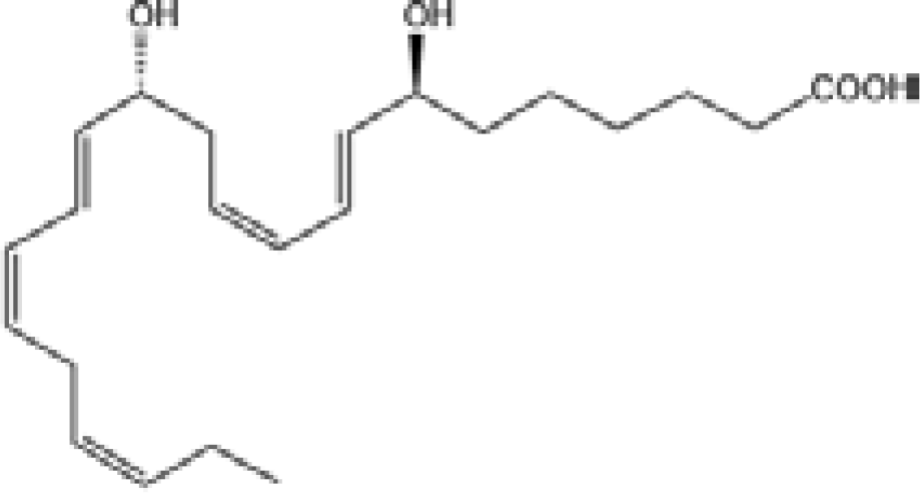	N/A	N/A
Resolvin D1 n-3 DPA(RvD1_n-3 DPA_)	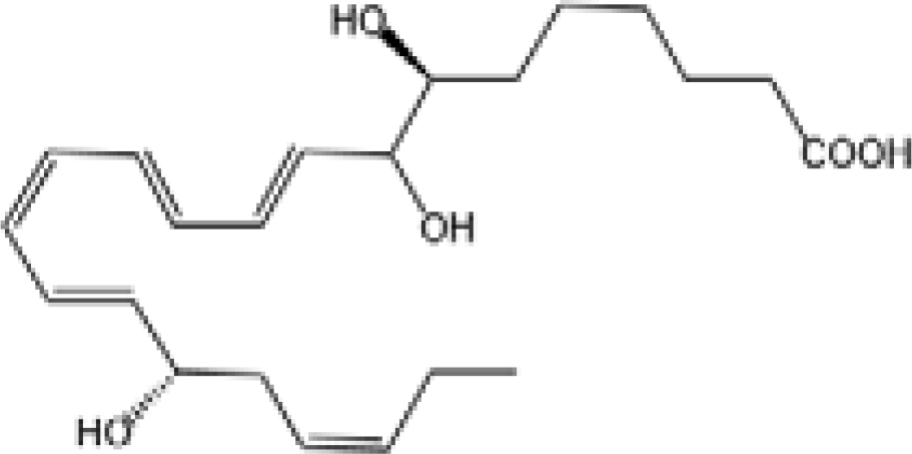	ALX/FPR2 and DRV1/GPR32 (135)	N/A
Resolvin D2 n-3 DPA (RvD2_n-3 DPA_)	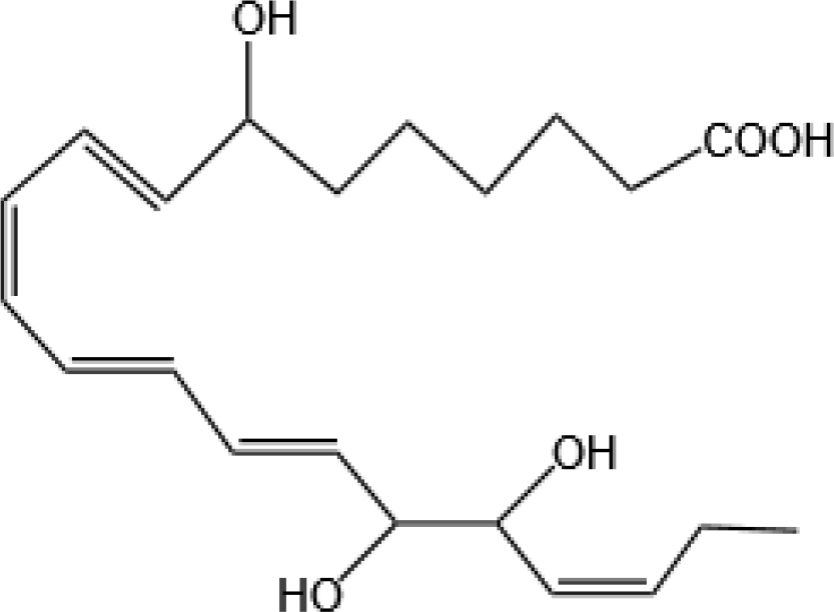	N/A	N/A
Resolvin D5 n-3 DPA (RvD5_n-3 DPA_)	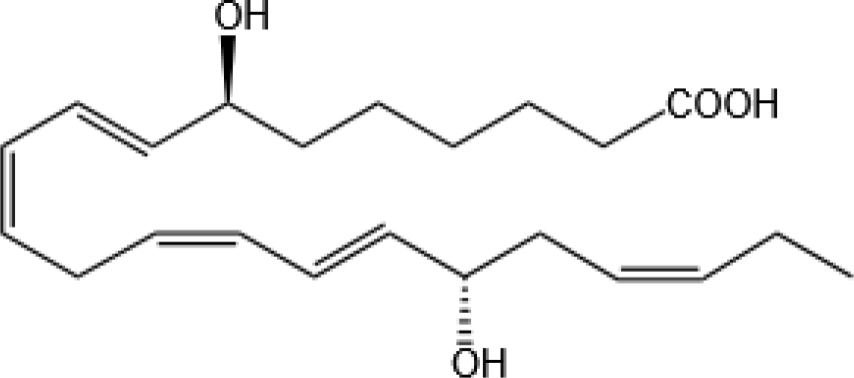	GPR101 and ALX/FPR2 (30)	YES (136)
Resolvin T1 n-3 DPA (RvT1 _n-3 DPA_)	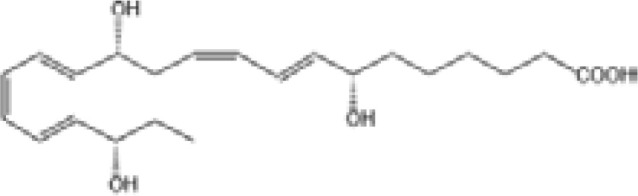	N/A	N/A
Resolvin T2 n-3 DPA (RvT2_n-3 DPA_)	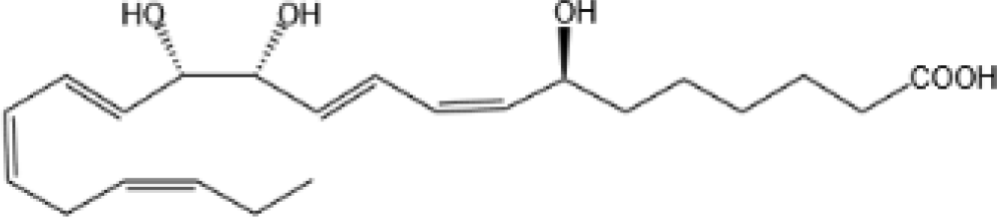	N/A	N/A
Resolvin T3 n-3 DPA (RvT3_n-3 DPA_)	unknown	N/A	N/A
Resolvin T4 n-3 DPA (RvT4_n-3 DPA_)	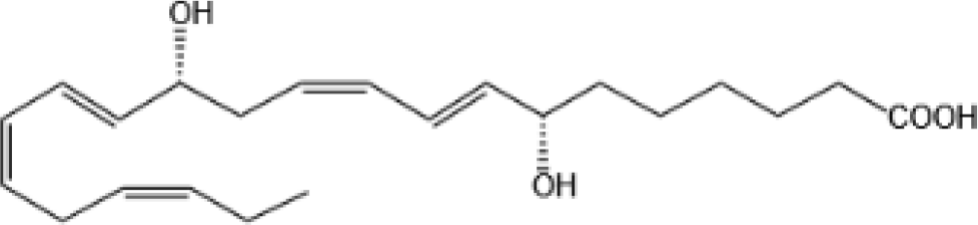	N/A	N/A
Resolvin Conjugates in Tissue Regeneration 1(RCTR1)	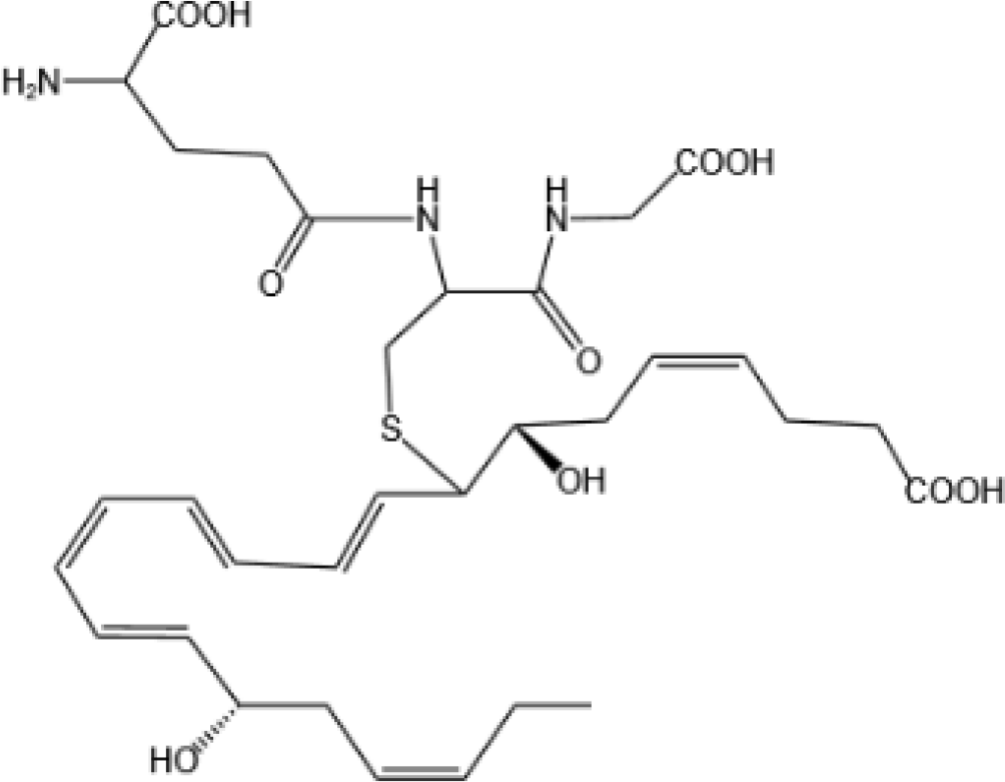	ALX/FPR2 (137)	N/A
Resolvin Conjugates in Tissue Regeneration 2(RCTR2)	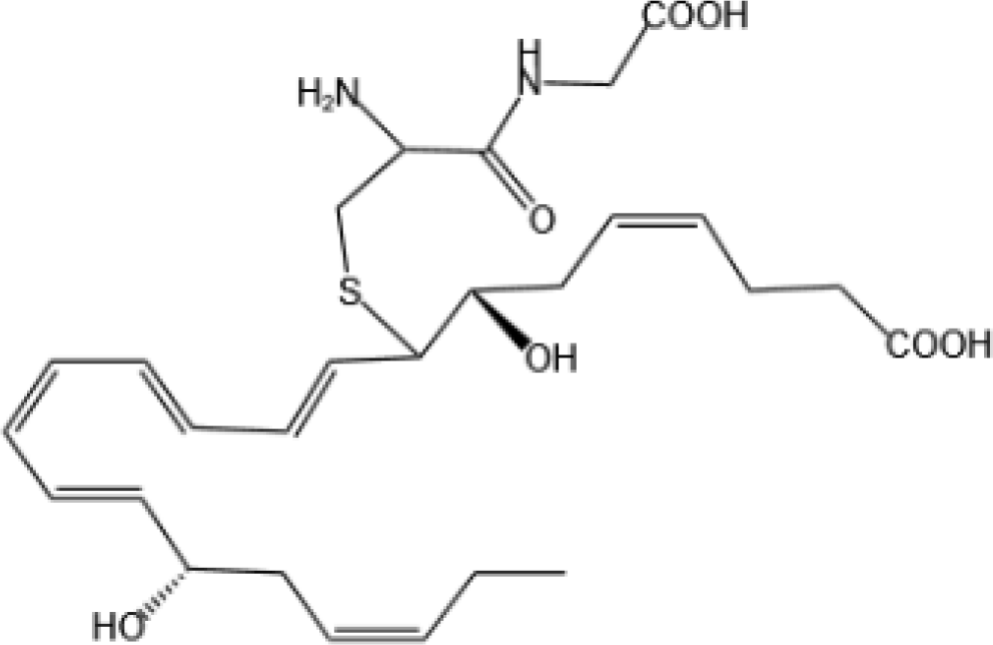	N/A	N/A
Resolvin Conjugates in Tissue Regeneration 3(RCTR3)	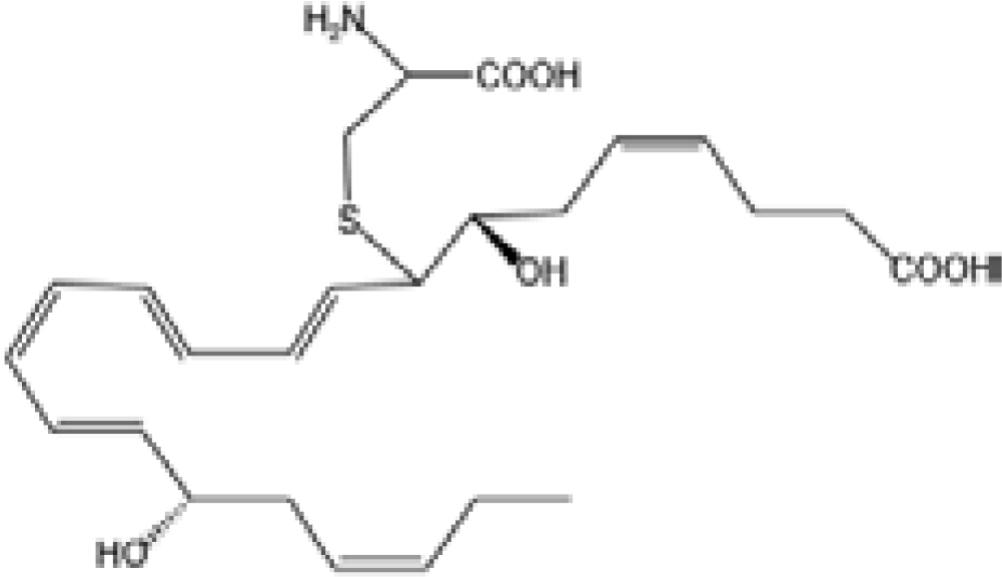	N/A	N/A
Aspirin-Triggered Resolvin D1(AT-RvD1)	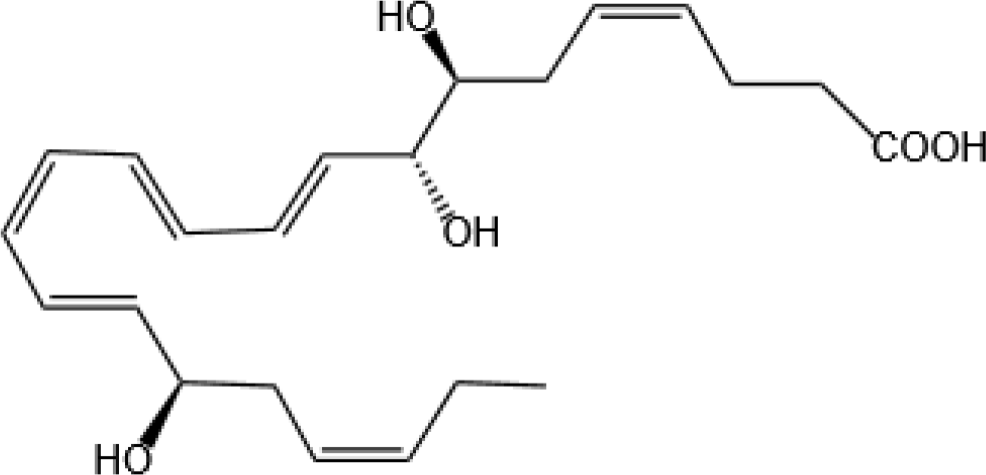	ALX/FPR2 (138)	YES (42)
Aspirin-Triggered Resolvin D2(AT-RvD2)	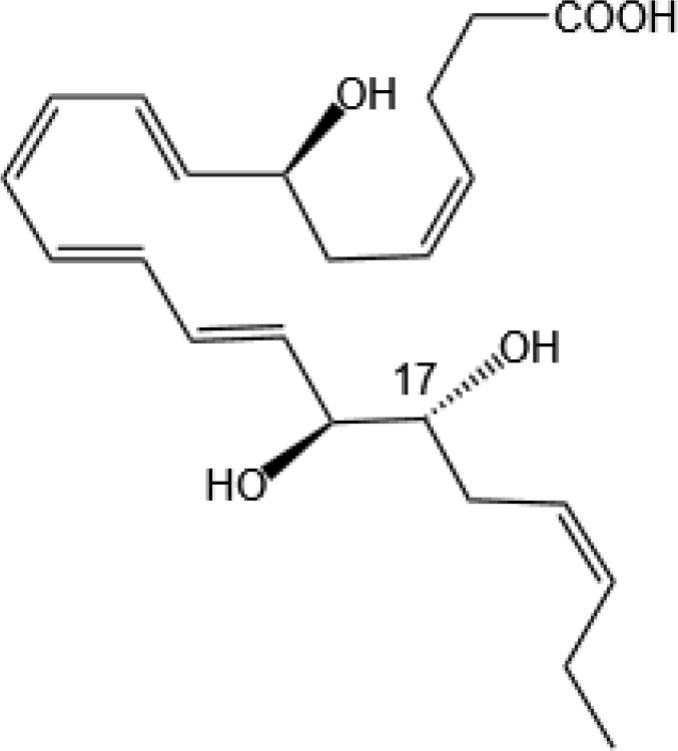	N/A	N/A
Aspirin-Triggered Resolvin D3(AT-RvD3)	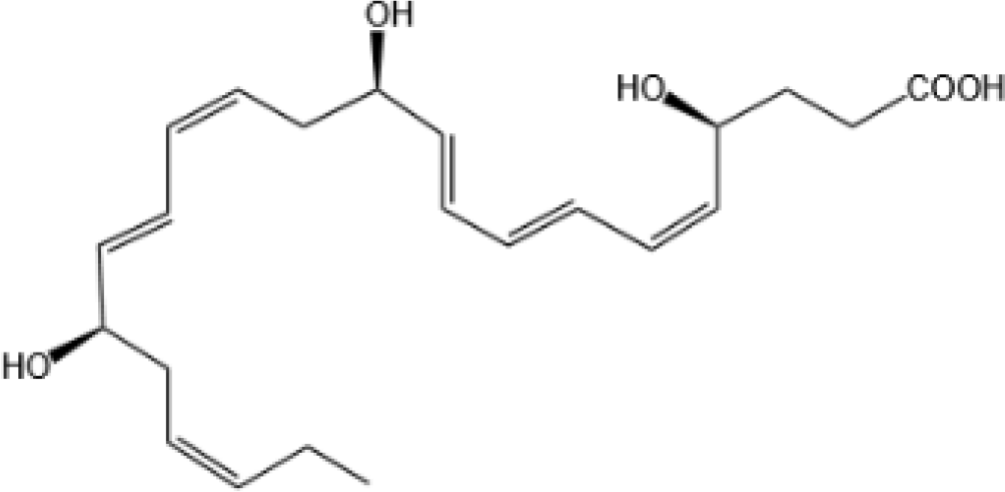	DRV1/GPR32 (139)	N/A
Aspirin-Triggered Resolvin D4(AT-RvD4)	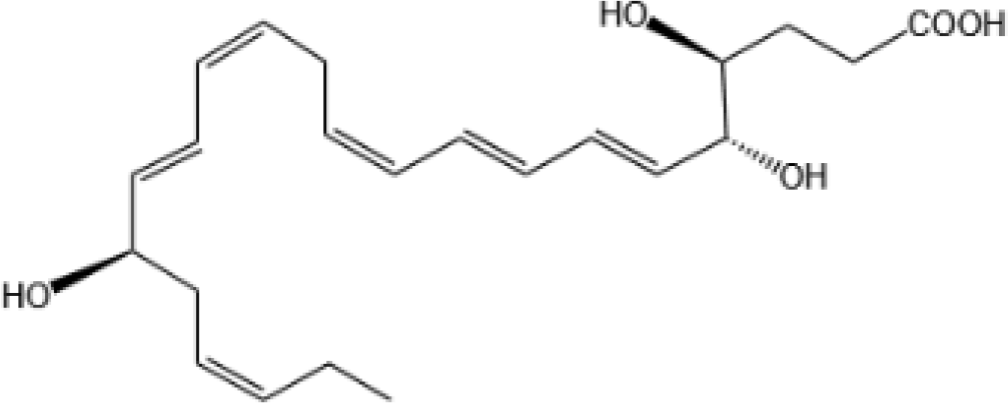	N/A	N/A
Resolvin E1(RvE1)	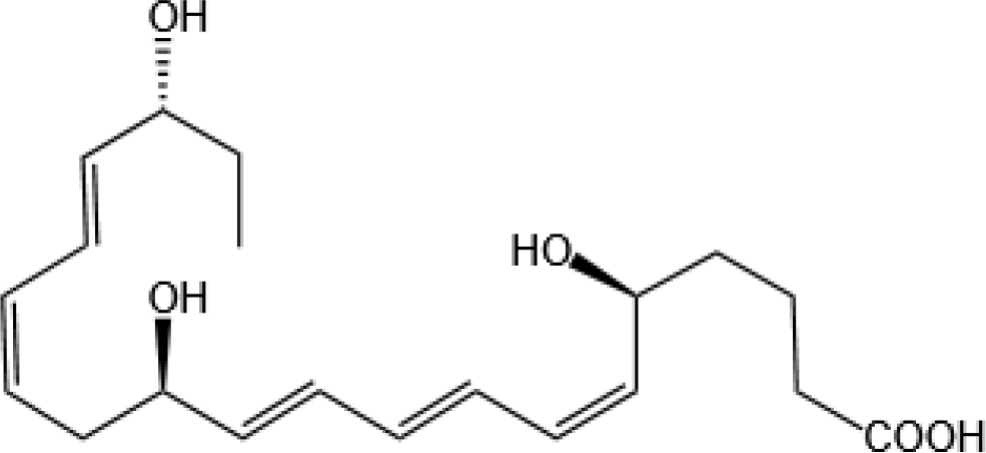	ChemR23 and BLT1 (134)	YES (113)
Resolvin E2(RvE2)	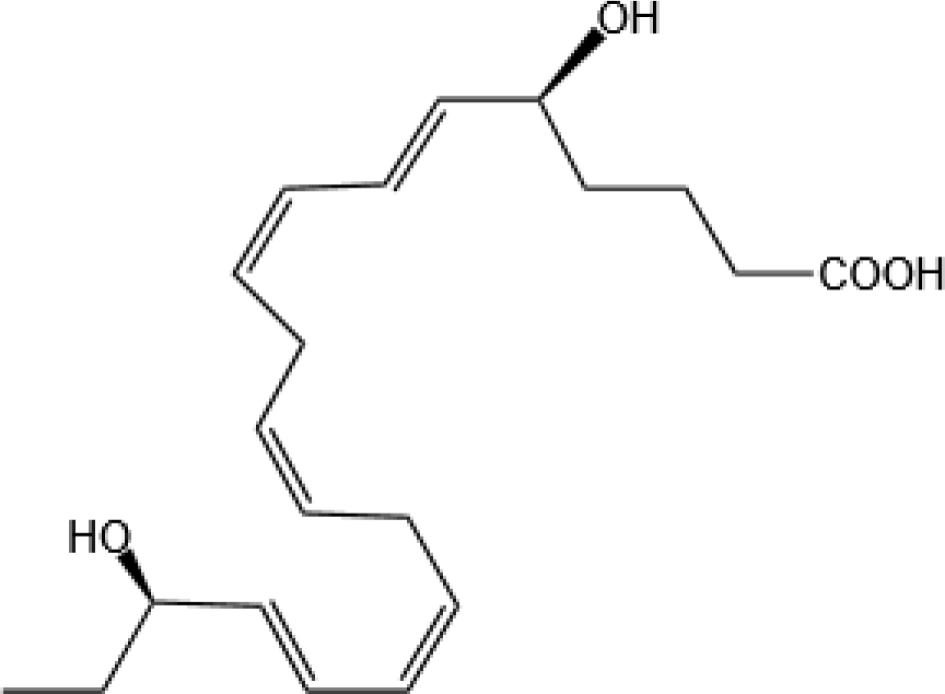	ChemR23 and BLT1 (23, 134)	N/A
Resolvin E3(RvE3)	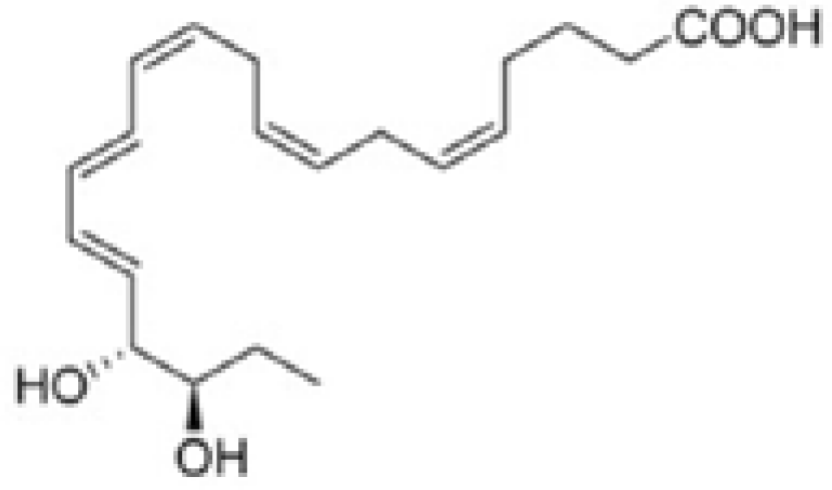	ChemR23 (140)	N/A
Resolvin E4(RvE4)	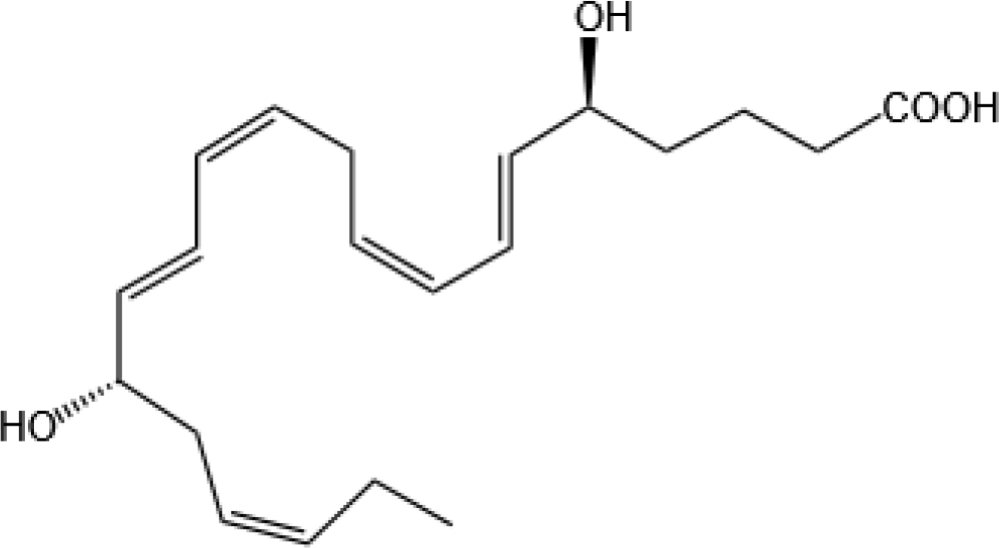	N/A	N/A
Protectins				
	Protectin D1 (PD1)	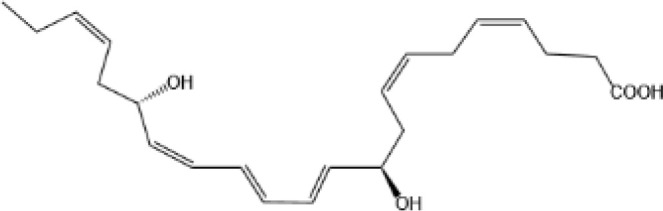	GPR37 (134)	YES (141)
	Protectin D2(PD2)	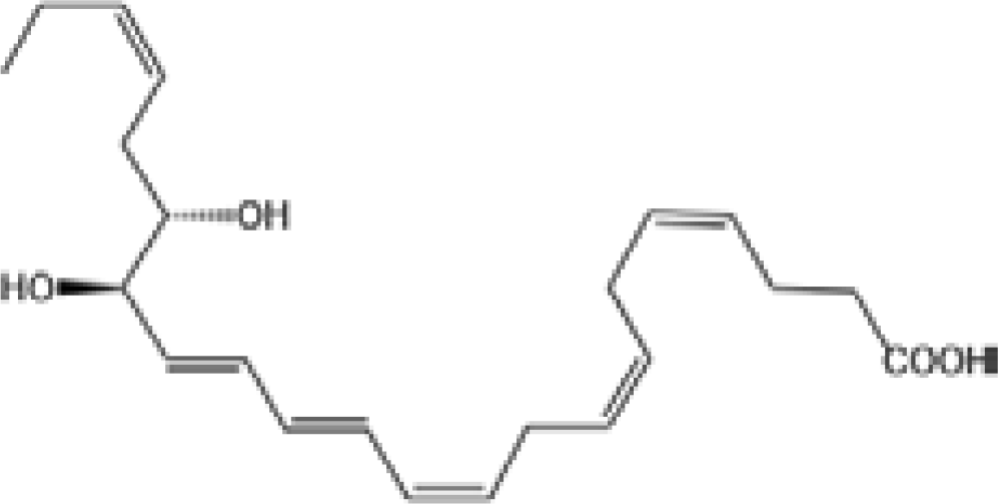	N/A	N/A
	Protectin DX(PDX)	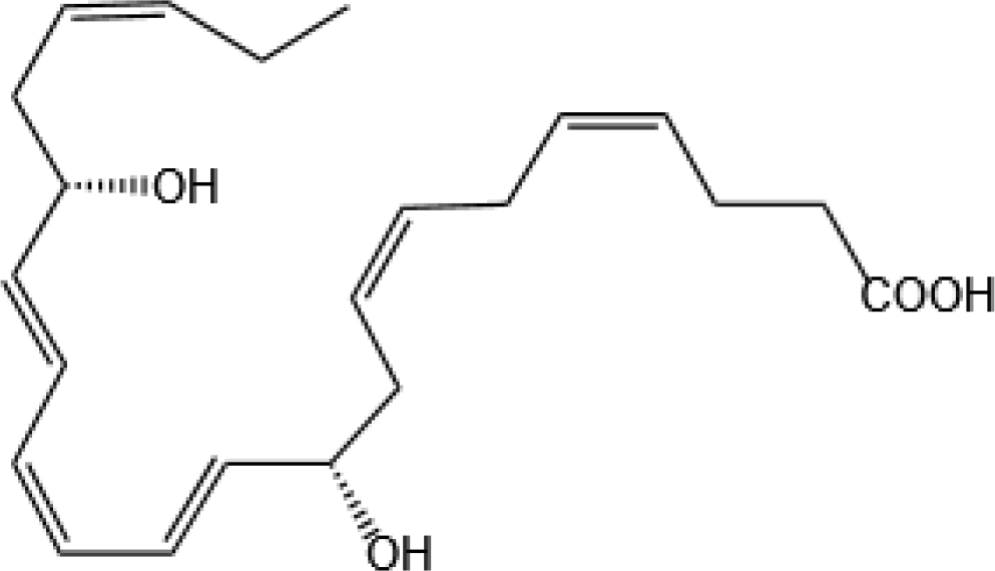	ALX/FPR2 (142) and GPR120 (31)	YES (109)
	Protectin D1 n-3 Docosapentaenoic Acid(PD1_n-3 DPA_)	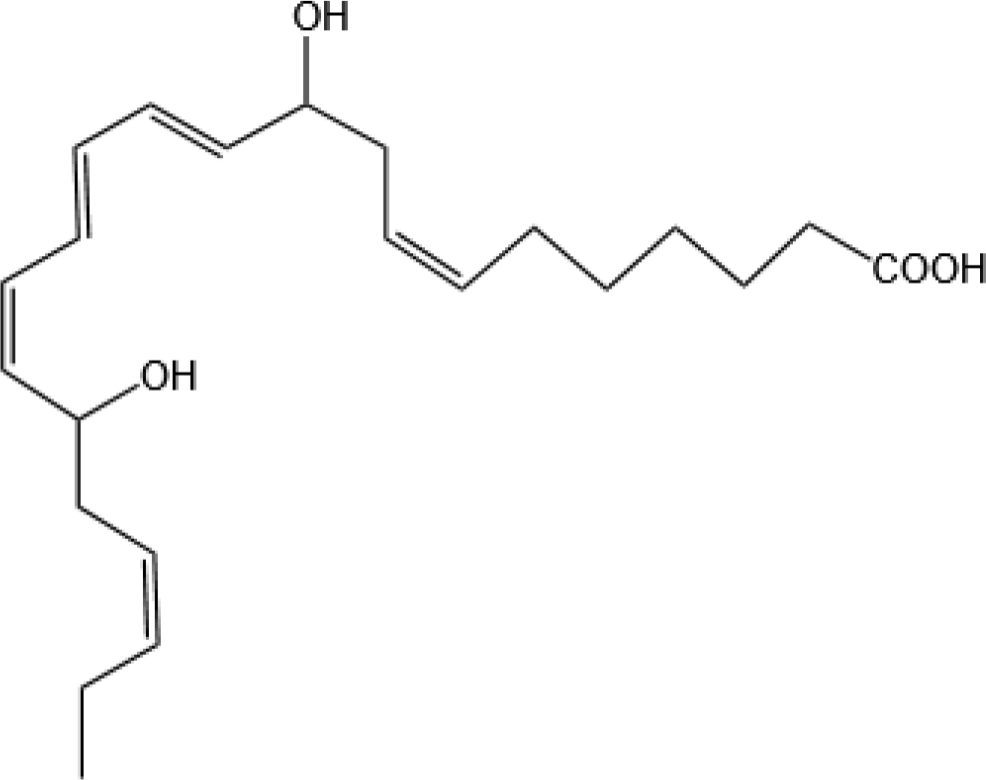	N/A	N/A
	Protectin D2 n-3 Docosapentaenoic Acid(PD2_n-3 DPA_)	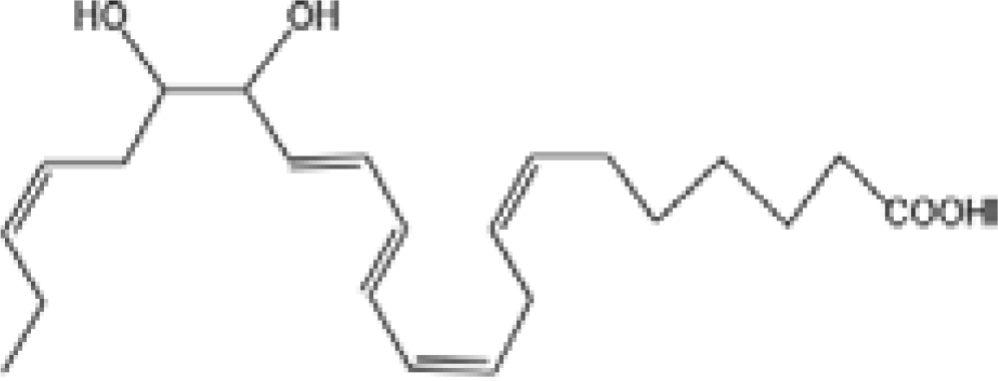	N/A	N/A
	Aspirin-Triggered Protectin D1(AT-PD1)	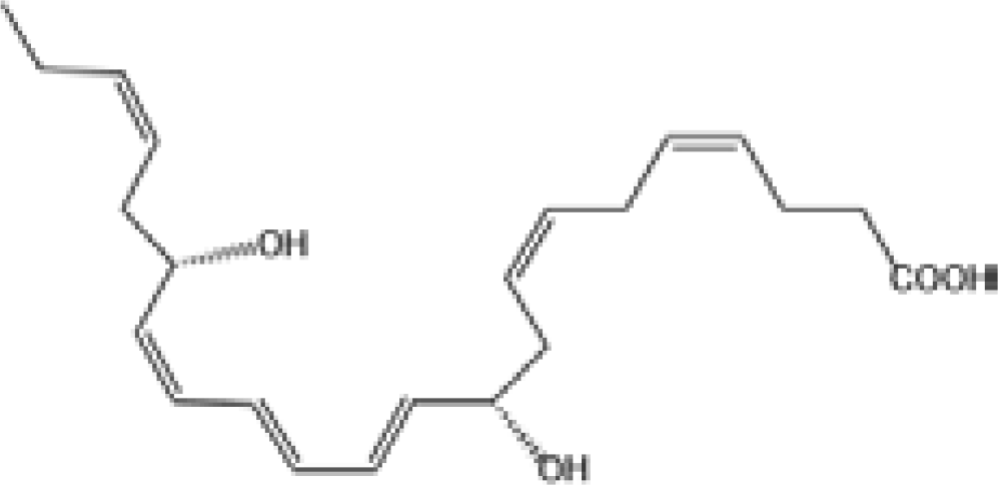	N/A	N/A
	Protectin Conjugates in Tissue Regeneration1(PCTR1)	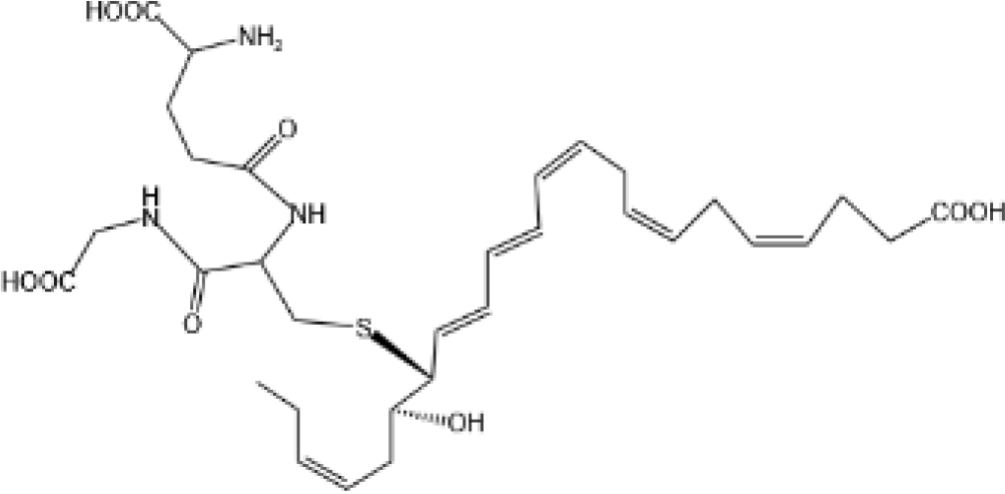	ALX/FPR2 (143)	YES (144)
	Protectin Conjugates in Tissue Regeneration2(PCTR2)	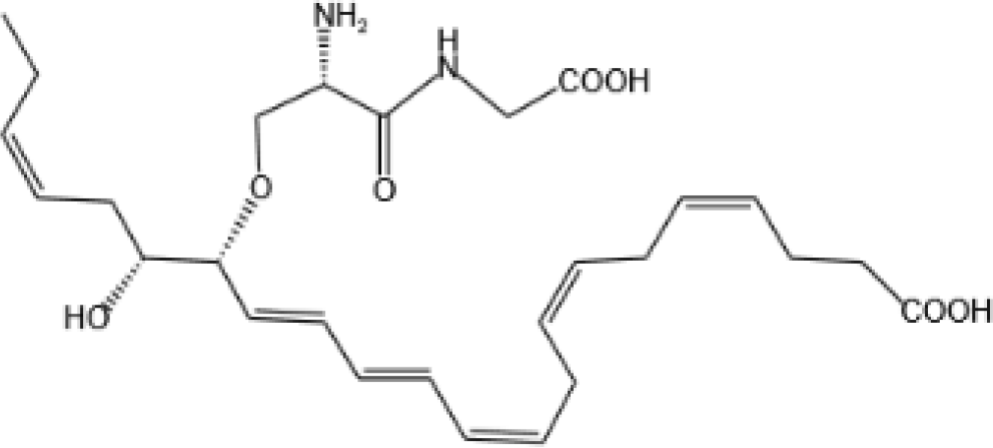	N/A	N/A
	Protectin Conjugates in Tissue Regeneration3(PCTR3)	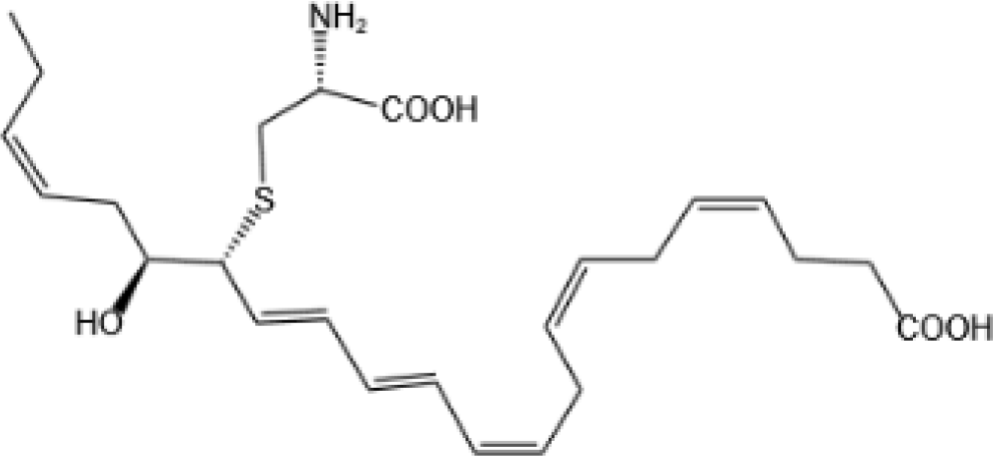	N/A	N/A
Maresins				
	Maresin 1(MaR1)	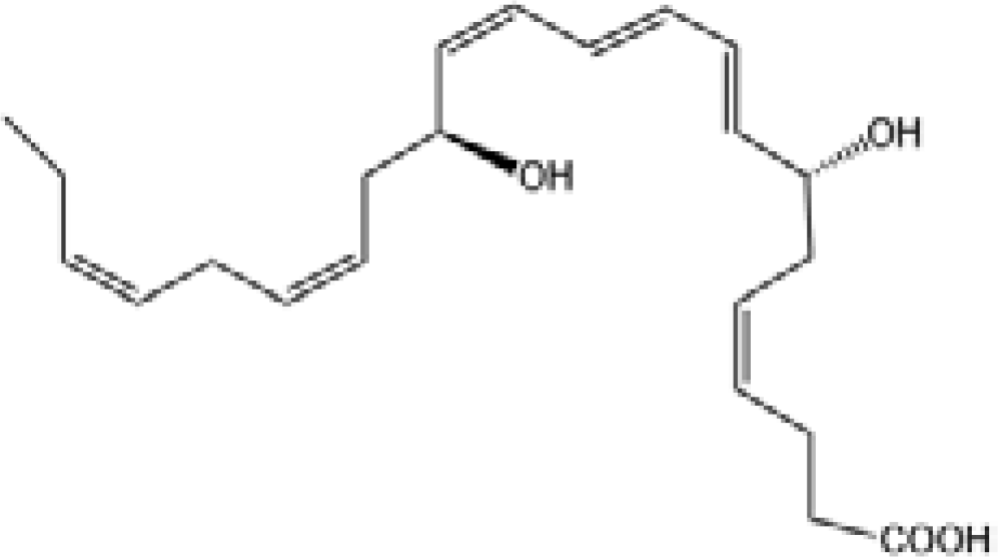	GPR37L1 (28) and RORα and LGR6 (32)	YES (28)
	Maresin 2(MaR2)	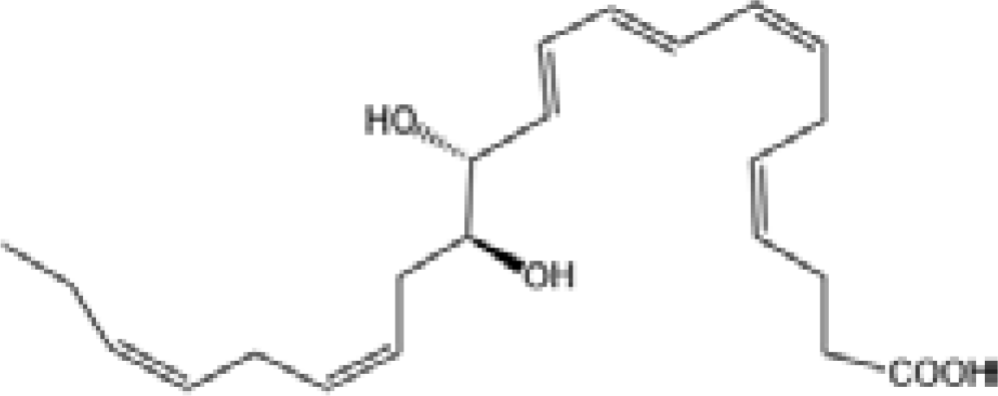	N/A	YES (60)
	Maresin 1 n-3 Docosapentaenoic Acid(MaR1_n-3 DPA_)	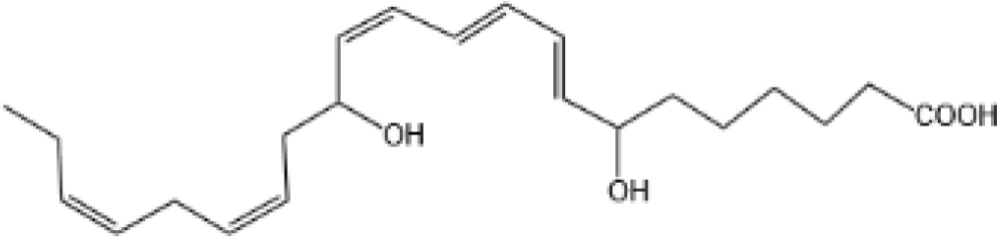	N/A	N/A
	Maresin 2 n-3 Docosapentaenoic Acid(MaR2_n-3 DPA_)	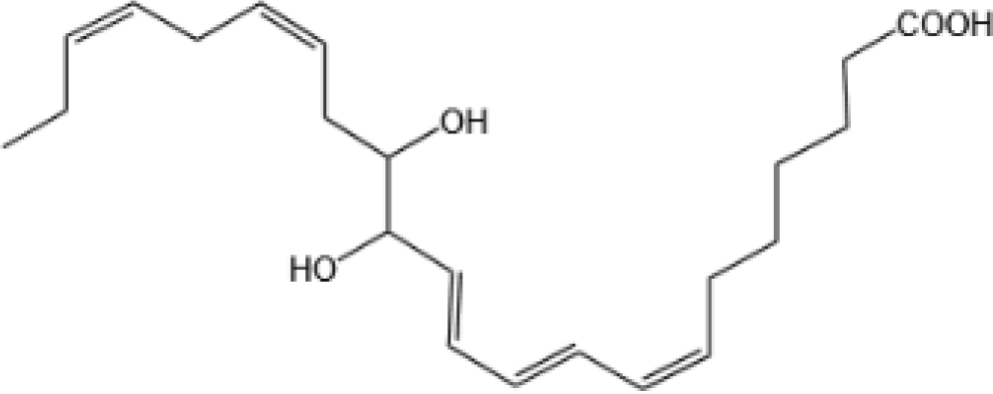	N/A	N/A
	Maresin 3 n-3 Docosapentaenoic Acid(MaR3_n-3 DPA_)	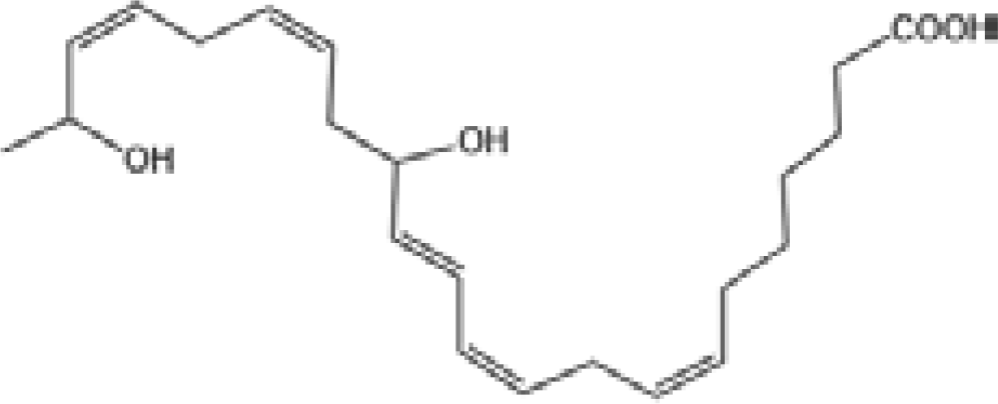	N/A	N/A
	Maresin Conjugates in Tissue Regeneration 1(MCTR1)	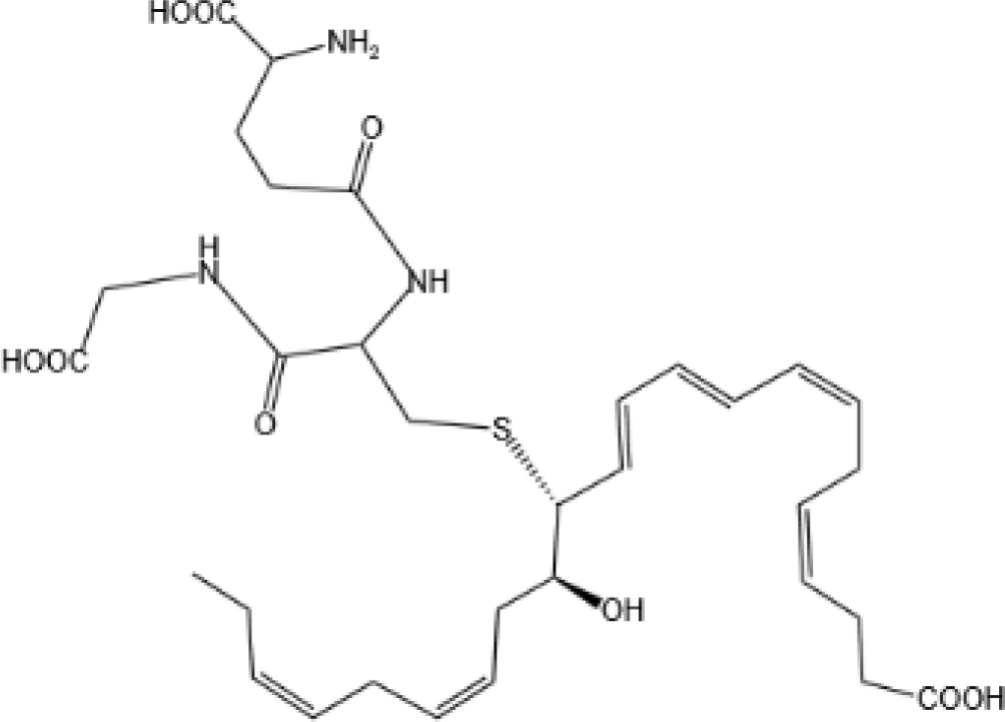	CysLT_1_ (33) and ALX/FPR2 (145)	N/A
	Maresin Conjugates in Tissue Regeneration 2(MCTR2)	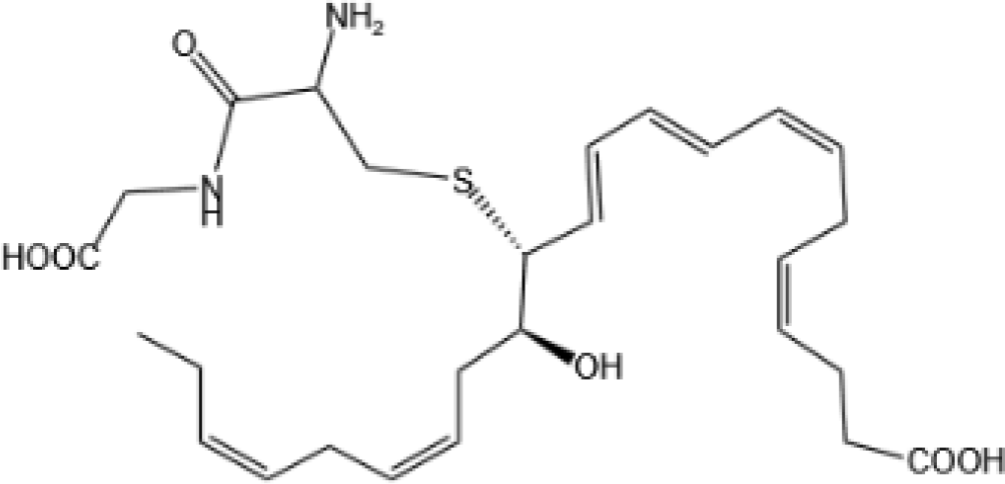	CysLT_1_ (33)	N/A
	Maresin Conjugates in Tissue Regeneration 3(MCTR3)	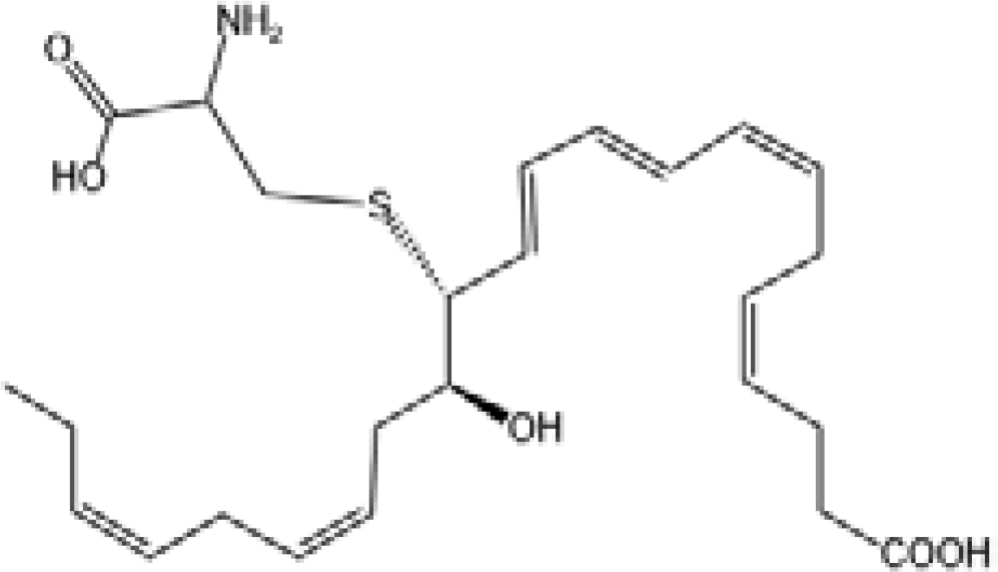	CysLT_1_ (33) and ALX/FPR2 (146)	N/A
lipoxin				
	Lipoxin A4(LXA4)	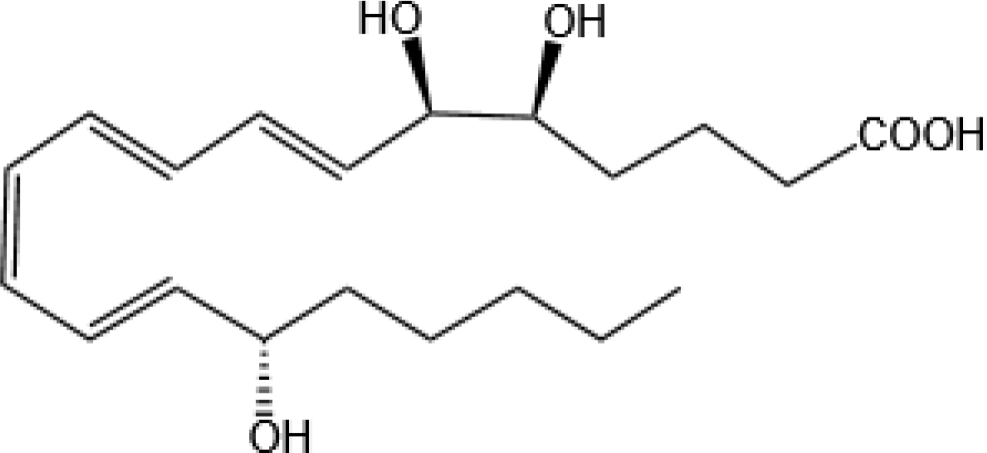	ALX/FPR2 (134) and AhR (34)	YES (56)
	Lipoxin B4 (LXB4)	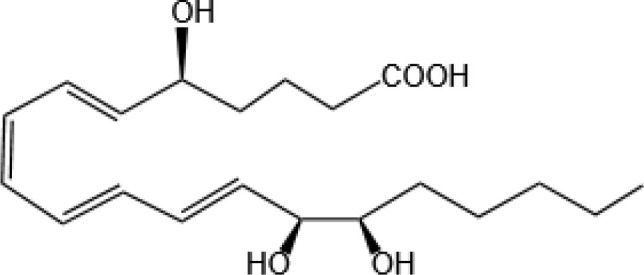	N/A	YES (57)
	Aspirin-Triggered Lipoxin A4(ATL)	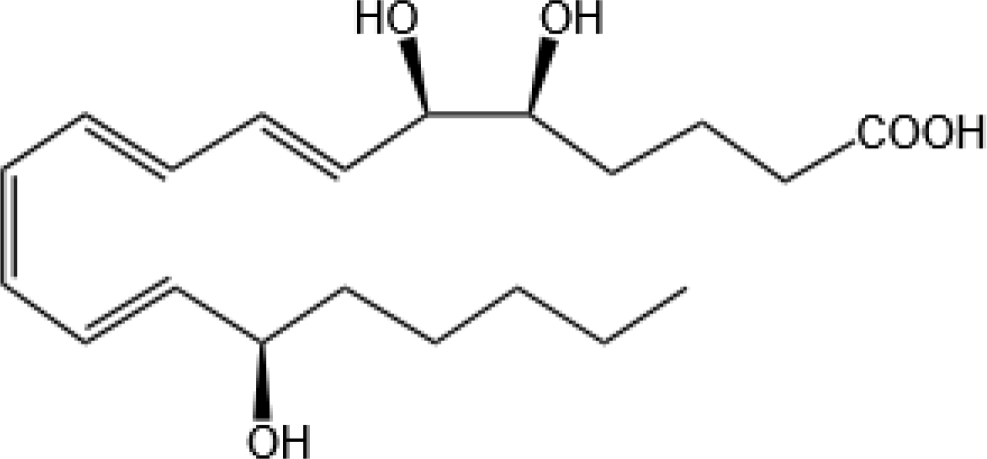	ALX/FPR2 (138)	YES (141)
	Aspirin-Triggered Lipoxin B4(AT-LXB4)	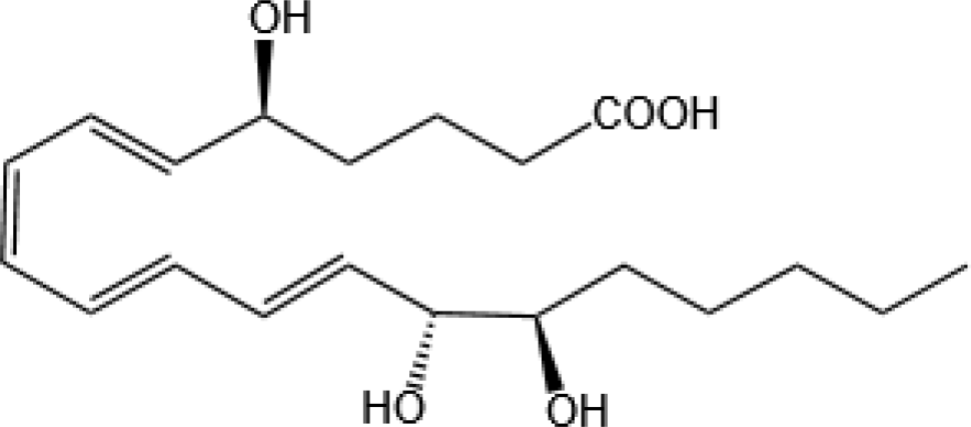	N/A	N/A

DRV1/GPR32: Resolvin D1 Receptor 1/G Protein-Coupled Receptor 32; SR-BI: scavenger receptor class B type 1; CysLT1: cysteinyl leukotriene 1; AhR: aryl hydrocarbon receptor N/A, Not Available.

The exploration of SPMs for pain relief is in its early stages, yet preclinical trials already indicate these compounds may become valuable therapeutic options. Notably, resolvins—unlike conventional analgesics such as morphine (35)—do not alter baseline pain sensitivity in healthy rodents, suggesting a favorable safety profile due to their targeted activity in pathological states (36–39). In preclinical models, SPMs demonstrate promising efficacy compared with traditional pain medicine. Resolvins outperformed gabapentin in alleviating neuropathic pain in the chronic constriction injury (CCI) model (40). In the formalin-induced inflammatory pain model, their effective doses were substantially lower than those required for morphine or the Cyclooxygenase-2 (COX-2) inhibitor NS-398 (36). Compared with standard anti-inflammatory drugs, only DEXA alleviated pain evoked by both 500 μg and 100 μg carrageenan, whereas INDO and CX were effective only against the high-dose stimulus. In contrast, RvD1 and RvE1 reduced nociception at both concentrations, demonstrating that the resolvins possess a broader therapeutic spectrum of anti-inflammatory and analgesic potential than traditional analgesics (41). Administration of 17(R)-HDoHE attenuates Complete Freund’s Adjuvant (CFA)-induced nociception. This analgesic potency is superior to that of clinically standard analgesics, including indomethacin, morphine, gabapentin, and dexamethasone (42) in the rat model. These findings suggest that SPMs may offer improved therapeutic efficacy compared to traditional analgesics, with the added advantage of reduced off-target effects. However, the clinical translation of SPMs is challenged by their metabolic instability, as they can be rapidly inactivated and degraded *in vivo*. Consequently, developing more stable SPM analogs or advanced delivery systems is crucial to realizing their therapeutic potential (26, 43). Studies have demonstrated that the metabolically stable RvE1 analog, 19-(p-fluorophenoxy)-RvE1 (19-pf-RvE1), produces potent and sustained antinociceptive effects by resisting rapid inactivation, a limitation associated with native RvE1 (36). This review synthesizes current evidence on the anti-nociceptive effects of SPMs and examines their mechanisms of action, aiming to inform future translational research and therapeutic development.

## 2 Anti-nociceptive effects of SPMs

### 2.1 Neuropathic pain

Neuropathic pain arises from lesions or diseases affecting the somatosensory nervous system (44), and is clinically characterized by spontaneous pain, hyperalgesia (heightened pain sensitivity to noxious stimuli), and paresthesia (abnormal sensations such as tingling or numbness). These symptoms often manifest as heterogeneous sensory abnormalities, complicating both diagnosis and management. Current treatment options for neuropathic pain—including antidepressants, anticonvulsants, and opioids—are limited by variable efficacy, adverse side effects, and high healthcare costs. While these pharmacological interventions may transiently alleviate symptoms, they often fail to modify the underlying pathophysiology driving pain chronification (45). This gap underscores the urgent need for therapies that target disease mechanisms rather than merely suppressing symptoms. Case series studies suggest that omega-3 fatty acids may benefit the management of patients with neuropathic pain (46).

Preclinical studies highlight the importance of timing in SPM administration for sustained anti-nociceptive efficacy. Timing-dependent effects are also observed in the CCI model: early post-surgical administration of RvD2 for 3 days induced robust analgesia lasting ≥4 days, whereas delayed RvD2 treatment yielded only transient relief (47). In the spinal nerve ligation model, intrathecal RvE1 administered 3 weeks post-injury rapidly reduced mechanical hypersensitivity within 1 hour, though effects waned by 3 hours (48). Notably, prophylactic PD1 administered during surgery completely prevented CCI-induced mechanical hyperalgesia for 4 weeks. When delivered at 3, 24, and 48 hours post-surgery, PD1 blocked neuropathic pain development, while injections initiated 2 weeks post-surgery alleviated established neuropathic pain for >3 hours (40). PD1 alleviates mechanical and thermal hyperalgesia in the CCI model. In contrast, its precursor DHA fails to produce significant analgesia, even at high doses, highlighting the necessity of SPM biosynthesis for therapeutic efficacy in neuropathic pain (40). In diabetic neuropathic pain models, 3-oxa-PD1n-3 DPA exerts remarkable anti-nociceptive effects within just one hour. Moreover, it has a lower effective dose than PD1 and PD1n-3 DPA. However, at a high dose of 300 pmol (i.t.), its anti-nociceptive effect duration is shorter than that of PD1n-3 DPA (49). In a rat postoperative pain model, daily intrathecal MaR2 (10 ng) administered on days 10, 12, 14, and 16 post-surgery reversed mechanical allodynia, with effects persisting until day 18 before diminishing by day 20 (50). Similarly, in mice, the same MaR2 dosing regimen (days 3, 5, 7, and 9 post-surgery) produced analgesia lasting until day 11, followed by a decline on days 12–13 (50). These findings underscore that SPMs exert time-sensitive therapeutic effects, with early intervention favoring prolonged efficacy (See **Table 2**).

**Table 2:** Anti-nociceptive effect of SPM in neuropathic pain model.

SPM molecules	Models	SPM administration routes	Effects	Relevant cells	Mechanisms	References
RvD1	NCLDH	IT (10 or 100 ng)	Mechanical pain↓	N/A	Regulate cytokines (TNF-α, IL-1β↓, TGF-β1, IL-10↑), and attenuate the expression of NF-κB/p65 and p-ERK in a dose-dependent manner.	(82)
	SNL	IP (10 or 100 ng)	Mechanical pain↓	Microglia	Reduce microglial activation; inhibit ERK/NLRP3/IL-1β signaling pathway through ALX/FPR2 receptor in spinal cord and DRG.	(79)
	SNI	IT (100 or 500 ng)	Mechanical pain↓	N/A	N/A	(128)
	SNI	IT (40 ng)	Mechanical pain and thermal pain↓	Microglia	Block microglial BDNF expression via ALX/FPR2, suppress BDNF/TrkB signaling, inhibit pro-inflammatory cytokines (TNF-α, IL-1β, and IL-6), down-regulate Bax and up-regulate Bcl-2 to exert neuroprotection.	(100)
	Paclitaxel	IP (5 μg/kg/day)	Mechanical and cold pain↓	Macrophage/ DRG neuron	Up-regulate IL-10 in macrophages through FPR2, and then IL-10 activates Nrf2-HO1 pathway in DRG neurons.	(147)
	Paclitaxel	IT (100 ng)	Mechanical pain↓	N/A	N/A	(25)
AT-RvD1	SNL	IP (10ng or 100ng)	Mechanical pain↓Thermal pain↓	Microglia	Inhibit the expression of NLRP3 inflammasome protein and its corresponding downstream pro-inflammatory factors (IL-18, IL-1β, and TNF-α) by up-regulating autophagy.	(98)
	Fibromyalgia-like	IT (80 ng)	Mechanical pain↓	N/A	N/A	(148)
	Fibromyalgia-like	IV (300 ng)/IT (80 ng)	Thermal pain↓			(148)
RvD2	CCI	IT (500 ng)/IV (5 μg)	Mechanical pain↓ Thermal pain↓	Astrocyte	Reduce spinal IL-17 secretion, CXCL1 release, and astrocyte activation.	(47)
	SCI	IT (50 ng/kg)	Mechanical pain↓Thermal and cold pain	Microglia	Target PTEN via the NF-κB–miR-155 axis to restore impaired autophagic flux, promote M2 anti-inflammatory polarization, and suppress TNF-α-induced microglial inflammation.	(106)
	Fibromyalgia-like	IV (300 ng)/IT (40 ng)	Mechanical pain↓ Thermal pain↓	N/A	N/A	(148)
	Paclitaxel	IT (100 ng)	Mechanical pain in both sexes↓	N/A	N/A	(25)
RvD3	SCI	IT (1 μg/20 μL)	Thermal pain↓	Macrophage	Lower spinal pro-inflammatory cytokines (TNF-α, IL-1β, IL-6) and chemokines (CCL-3, CCL-2); drive macrophage polarization toward the anti-inflammatory M2 phenotype.	(74)
RvD5	Paclitaxel	IT (100 ng)	Mechanical pain in males↓	N/A	N/A	(25)
RvE1	CCI	IT (100 ng)	Mechanical pain↓	Microglia	Inhibit microglial signal transduction (decrease synthesis and release of TNF-α).	(48)
	SNL	IT (100 ng)	Mechanical pain↓Thermal pain↓	N/A	N/A	(48)
PD1	DPNP	IT (90, and 300 pmol)	Mechanical pain↓	N/A	N/A	(49)
	CCI	Around the sciatic nerve (300 ng)/IV (600 ng)/IT (20, 100 or 500 ng)	Mechanical pain↓Thermal pain↓	Astrocyte /Microglia /Spinal neuron /Macrophage	Suppress spinal microglial/astrocyte reactivity, reduce IL-1β, CCL2 and p38/ERK phosphorylation, reshape synaptic plasticity; reverse DRG IB4 loss and activating transcription factor 3 (ATF-3) elevation, and block inflammatory macrophage infiltration.	(40)
	SNL	Around the sciatic nerve (300 or 900 ng)	Mechanical pain↓	Spinal neuron	Reshape synaptic plasticity.	(40)
	Axotomy/Amputation	Around the sciatic nerve (300 ng)	Phantom limb pain↓	N/A	N/A	(40)
	DPNP	Systemic (600 ng)	Mechanical pain↓	N/A	N/A	(40)
	NCLDH	IT (25 μM or 10 μL)	Mechanical pain↓Thermal pain↓	N/A	Regulate cytokine levels (TNF-α, IL-1β↓, TGF-β↑) by targeting the SIRT1/CGRP axis.	(108)
	Sciatic nerve crush	IV (600 ng)	Thermal and mechanical pain↓	Astrocyte /Microglia /Macrophage	Reduce the activation of microglia and astrocytes, downstream pro-inflammatory cytokines (IL-1β, IL-6, and TNF-α) in the spinal cord; prevent the infiltration of IBA-1+ macrophages in DRGs.	(149)
	Sciatic nerve crush	IT (100 ng)	Mechanical pain↓			(149)
PD1n-3 DPA	DPNP	IT (90, and 300 pmol)	Mechanical pain↓	N/A	N/A	(49)
3-oxa-PD1n-3 DPA	DPNP	IT (30, 90, and 300 pmol)	Mechanical pain↓	N/A	N/A	(49)
PDX	DPNP	IP (3 ng or 10 ng)	Mechanical pain↓	N/A	N/A	(83)
	NCLDH	IT (10 ng or 100 ng)	Mechanical pain↓Thermal pain↓	N/A	Regulate cytokines (IL-6, IL-1β↓, TGF-β1↑); promote autophagic flux (LC3B↑, P62↓) and AMPK signaling.	(109)
MaR1	Vincristine	IP (40 ng)	Mechanical pain↓	N/A	N/A	(59)
	SNI	Oral (50 μg/kg)	Mechanical pain↓	Astrocyte /Microglia	Reduce microglia and astrocyte activation; slightly increase M-CSF (in males).	(102)
	SNI	IT (100 ng)	Mechanical pain↓	Astrocyte	Increase astrocyte GLT-1 activity and reduce spinal EPSC by activating GPR37L1.	(28)
	SNL	IT (100 ng)	Mechanical pain↓Thermal pain↓	Astrocyte /Microglia	Reduce activation of microglia and astrocyte; inhibit spinal NF-κB activation and nuclear translocation, and cytokine production (TNF-α, IL-1β, and IL-6); restore synaptic integrity.	(111)
	NCLDH	IT (100 ng)	Mechanical pain↓Thermal pain↓	N/A	Reduce the level of pro-inflammatory cytokine (IL-1β, IL-18); prevent NLRP3 inflammasome activation.	(104)
	NCLDH	IT (10 ng or 100 ng)	Mechanical pain↓Thermal pain↓	N/A	Inhibit NLRP3 inflammasome-induced pyroptosis via NF-κB signaling and downregulate inflammatory cytokine levels.	(103)
	CCI	IT (10 ng or 100 ng)	Mechanical pain↓Thermal pain↓	N/A	N/A	(150)
MaR2	CCI-ION	IT (10 ng)	Thermal and mechanical pain↓	TG neuron	Reduce c-fos-positive neurons and CGRP+ activated (nuclear pNFkB) neurons.	(50)
LXA4	SCI	IT (300 pmol)	Mechanical pain↓	Microglia	Regulate microglia activation and TNF-α release through ALX/FPR2 receptor.	(101)
	NCLDH	IT (10ng or 100ng)	Mechanical pain↓	N/A	Regulate cytokines (TNF-α, IL-1β↓, TGF-β1, IL-10↑); reduce the expression of NF-κB/p65, p-ERK, and p-JNK.	(105)
	CCI	IT (1μg)	Mechanical pain↓	N/A	N/A	(112)
ATL	CCI	IT (200ng)/IV (10μg/kg or 40μg/kg)	Mechanical pain↓	Astrocyte	Block JAK2-STAT3 signaling pathway by acting on astrocyte surface ALX receptors, upregulate SOCS3 and decrease inflammatory factors (IL-1, IL-6, and TNF-α↓).	(112)
	CCI	IT (200 ng)	Thermal pain↓	N/A	Inhibit NALP1 inflammasome activation, caspase-1 cleavage, and IL-1β maturation.	(151)
	DPNP	IT (1,3,10 or 30 ng)	Mechanical pain↓	N/A	N/A	(152)

CCI, chronic constriction injury; CCI-ION, chronic constriction injury of the rat’s infraorbital nerve; DPNP, diabetic peripheral neuropathic pain; IP, intraperitoneal; IT, intrathecal; IV, intravenous; NCLDH, noncompressive lumbar disk herniation; SCI, spinal cord injury; SNI, spared nerve injury; SNL, spinal nerve ligation. N/A, Not Available.

### 2.2 Inflammatory pain

Inflammatory pain arises as a consequence of immune system activation, a protective physiological response to tissue injury or infection. However, this response can paradoxically amplify pain through the release of pro-inflammatory mediators such as cytokines, chemokines, and prostaglandins (51). These mediators act directly on nociceptors (pain-sensing neurons), lowering their activation thresholds and increasing spontaneous firing rates. This process drives peripheral sensitization—heightened sensitivity at the injury site—and central sensitization, a maladaptive plasticity in the spinal cord and brain that sustains pain even after tissue healing (52). Together, these mechanisms transform acute protective inflammation into chronic, pathological pain states.

Multiple lines of evidence demonstrate that SPMs attenuate CFA-induced nociception (**Table 3**). RvD1 reduced mechanical hypersensitivity with efficacy similar to AP18, a transient receptor potential ankyrin 1 (TRPA1) channel antagonist (53). Repeated AT-RvD1 administration (twice daily for 4 days) further suppressed the development of CFA-induced mechanical hyperalgesia, suggesting cumulative benefits with prolonged treatment (42). RvD2, administered 3 days post-CFA, diminished thermal hyperalgesia and mechanical allodynia for >3 hours (38). RvE1, delivered on day 3 post-CFA, attenuated thermal hyperalgesia within 15 minutes, though effects were transient (36). In CFA-induced thermal hyperalgesia, RvE1 achieves comparable pain relief at doses 10,000-fold lower than those required for DHA and EPA, underscoring the superior potency of SPMs in inflammatory settings (36). Both RvD1 and RvE1 can alleviate nociception induced by CFA, but RvE1 is twice as potent as RvD1 in reducing paw withdrawal responses to mechanical stimuli at 3 h and 2 h post-treatment (41). 10 ng of PD1 induced a rapid reduction in thermal hyperalgesia within 20 minutes, lasting for 2 hours, while 1 ng of PD1 sustained its anti-nociceptive effect for 40 minutes (37).

**Table 3:** Anti-nociceptive effect of SPM in inflammatory pain model.

SPM molecules	Models	SPM administration routes	Effects	Relevant cells	Mechanisms	References
RvD1	MSU	IT (3 ng)/IP (3 ng)	Mechanical pain↓	DRG neuron /Macrophage /Leukocyte	Reduce total leukocyte recruitment; inhibit IL-1β production and CGRP release; block leukocyte accumulation around CGRP-positive nerve fibers in the knee joint; suppress IL-1β maturation, ASC protein expression and NF-κB activation in macrophages.	(54)
	CFA	IPL (20 ng)	Mechanical pain↓	HEK cells expressing TRP channels	Inhibit TRPA1, TRPV3 and TRPV4.	(53)
	EAP	IP (2.5 μg/kg, 5 μg/kg or 10 μg/kg)	Tactile pain↓	N/A	Inhibit oxidative stress and NLRP3 inflammasome activation through the Nrf2/HO-1 pathway, thereby reducing inflammatory cell infiltration in prostate tissue.	(5)
	CP	IT (100 ng/kg)	Mechanical pain↓	Spinal neuron	Decrease spinal dorsal horn neuronal NMDA receptor NR1 and NR2B subunit phosphorylation; downstream pro-inflammatory cytokines (IL-1β, IL-6 and TNF-α).	(39)
	burn injury	IP (300 ng)	Mechanical pain↓	Astrocyte /Microglia	Inhibit BDNF/TrkB signaling in the spinal dorsal horn, activation of microglia and astrocyte, and downregulate p38 MAPK in the microglia.	(99)
	carrageenan	IPL (20 ng)	Thermal pain↓	N/A	Reduce edema, neutrophil infiltration, and the expression of pro-inflammatory cytokine (TNF-α, IL-1β, IL-6) and chemokines (MCP-1, MIP-1α).	(36)
	carrageenan	IPL (285, 570 or 1140 pmol)	Mechanical pain↓	N/A	Inhibit inflammatory mediators such as 5-HT, SP, and PGE2.	(41)
	Capsaicin/Formalin	IPL (20 ng)	Acute nociception induced by formalin↓ No effect on pain induced by capsaicin	DRG neuron	Inhibit TRPV activation; reduce neurogenic inflammation via modulation of TRP channels.	(53)
17(R)-RvD1	carrageenan	IT (3 μg or 300 μg)	Mechanical pain↓	Astrocyte	Reduce TNF release in cerebrospinal fluid and astrocytes, and suppress TNF-induced ERK activation in astrocytes.	(56)
AT-RvD1	AIA	IP (100 ng or 300 ng)	Mechanical pain↓	N/A	Lower pro-inflammatory cytokine levels (TNF-α, IL-1β).	(42)
RvD2	CFA	IT (10 ng)	Thermal pain↓Mechanical pain↓	Spinal neuron	Induce spinal Synaptic plasticity.	(38)
	carrageenan	IPL (10 ng)	Thermal pain↓ Mechanical pain↓	DRG neuron	Inhibit TRPV1 and TRPA1.	(38)
	Interstitial cystitis/bladder pain syndrome	IT (100 ng)	Mechanical pain↓	DRG neuron	Activate GPR18 to inhibit TRPV1 channel activity.	(27)
	Formalin	IT (0.01 ng, 0.1 ng or 1 ng)	The second phase of pain ↓ No effect on the first phase	Spinal neuron	Inhibit TRPV1 and TRPA1 channels; block inflammation-induced synaptic plasticity and reverse LTP in the spinal cord.	(38)
	Capsaicin/AITC	IPL (10 ng)	Nocifensive behaviors↓	DRG neuron	Antagonize TRPV1 and TRPA1; reduce glutamate release from the presynaptic terminals of primary afferents; inhibit Ca^2+^ influx and neuropeptide release.	(38)
RvE1	CFA	IT (10 ng)	Thermal pain↓	DRG neuron /spinal neuron	Blockade of TRPV1- and TNF-α-mediated potentiation of excitatory synaptic transmission; blockade of TNF-α–evoked NMDAR hyperactivity.	(36)
	carrageenan	IPL (20 ng)	Thermal pain↓	Neutrophil	Reduce edema, neutrophil infiltration, and the expression of pro-inflammatory cytokines (TNF-α, IL-1β, IL-6) and chemokines (MCP-1, MIP-1α).	(36)
	carrageenan	IPL (RvE1: 57, 285 or 570 pmol)	Mechanical pain↓	N/A	Inhibit inflammatory mediators such as 5-HT, SP, and PGE2.	(41)
	Formalin	IT (0.3 or 1.0 ng)	The second phase of pain ↓ No effect on the first phase	DRG neuron /primary afferent terminals in the spinal cord	Block TNF-α/TRPV1 signaling via ChemR23 to suppress ERK phosphorylation and reduce presynaptic glutamate release.	(36)
	Formalin	IPL (20 ng)	Licking and flinching behaviors↓	N/A	N/A	(36)
PD1	CFA	IT (10 ng)	Thermal pain↓	N/A	Inhibit TRPV1 and the downstream AC/PKA/ERK pathway via activation of Gαi-coupled GPCR; block spinal LTP.	(37)
	TNF-α	IT (10 ng)	Thermal pain↓Mechanical pain↓	N/A	Block TNF-α-TRPV1-glutamate release pathway.	(37)
	Capsaicin	IT (10 ng)	Nocifensive licking behavior↓	Spinal cord neuron /Microglia /TRPV1+ presynaptic terminal	Inhibit TRPV1 current via Gαi-coupled GPCRs; block presynaptic glutamate release and ERK/PKA signaling pathways.	(37)
	Capsaicin	IPL (20 or 200 ng)	Nocifensive licking behavior↓	Peripheral sensory neuron	Inhibit TRPV1 channel function; reduce neurogenic inflammation and CGRP release.	(37)
	Formalin	IT (0.1, 1 or 10 ng)	The second phase of pain ↓ No effect on the first phase	Spinal cord lamina II excitatory neuron (vGluT2+) /presynaptic TRPV1+ terminal	Inhibit TRPV1 currents via Gαi-coupled GPCRs; block TNF-α-evoked glutamate release and ERK signaling.	(37)
MaR1	Mouse K/BxN serum metastasis	IP (100 ng)	Mechanical pain↓	DRG neuron/Macrophage	Inhibit TRPV1-mediated calcium influx and CGRP release via activation of Gαi-coupled GPCR; suppress pro-inflammatory cytokines; reduce the expression of miR-155.	(55)
	OA	IP (500 ng)	Mechanical pain↓	DRG neuron /Macrophage /satellite glial cell	Activate RORA receptors; inhibit TRPV1 channels; decrease calcium influx; inhibit inflammatory factors; decrease CGRP expression and macrophage activation.	(153)
	carrageenan	IT (10 ng)	Mechanical pain↓Thermal pain↓	Neutrophil /Macrophage	Inhibit NF-κB pathway activation; reduce production of TNF-α and IL-1β.	(92)
	CFA	IT (10 ng)	Mechanical pain↓Thermal pain↓	DRG neuron /astrocyte /Microglia	Inhibit CGRP release; reduce production of TNF-α and IL-1β; inhibit NF-κB pathway activation.	(92)
	Zymosan	Topical application to the shaved vulvar skin. (1 μg/30 μL/day)	Mechanical pain↓	Vulvar fibroblast	Reduce IL-6 and PGE2	(58)
	Capsaicin	IPL (10 ng)	Spontaneous pain↓	DRG neuron	Inhibit TRPV1 currents via Gαi-coupled GPCRs; reduce capsaicin-induced inward currents.	(59)
MaR2	LPS	IP (3, 10 or 30 ng)	Mechanical and thermal pain↓	DRG neuron	Inhibit TRPV1 and TRPA1 channels activation and inhibit CGRP release.	(60)
	Formalin	IT (10 ng)	Both the first and second phases of nociceptive responses↓	TG neuron	Inhibit neuronal activation; reduce CGRP release and p-NFκB signaling; modulate TRP channels indirectly.	(50)
	Capsaicin	IP (30 ng)	Overt pain-like behaviors↓	Systemic macrophage /DRG neuron	Modulate systemic immune response; inhibit TRPV1 activation and neuroinflammation.	(60)
	AITC	IP (30 ng)	Overt pain-like behaviors↓	Systemic macrophage /DRG neuron	Inhibit TRPA1-mediated signaling; reduce oxidative stress and cytokine release.	(60)
	Bothrops jararaca venom	IPL (0.3 ng or 1 ng)	Mechanical pain↓ Thermal pain↓	Neutrophil/Macrophage	Reduce myeloperoxidase activity, reverse oxidative stress, and decrease pro-inflammatory cytokine expression.	(154)
LXA4	carrageenan	IT (0.3 μg or 1 μg)	Mechanical pain↓	Astrocyte	Reduce TNF release via FPR2/ALX receptors.	(56)
	CCD	IT (10 ng or 100 ng)	Mechanical pain↓ Thermal pain↓	DRG neuron	Inhibit NF-κB activation and upregulation of pro-inflammatory cytokines (TNF-α, IL-1β and IL-6).	(81)
	carrageenan	IT (0.3 nmol)	Thermal pain↓	Astrocyte	Activate the ALXR receptor and inhibit ERK/JNK phosphorylation.	(57)
	Arthritis	IP (0.1, 1, or 10 ng)	Mechanical pain↓ Thermal pain↓	DRG neuron /Macrophage	Inhibit NF-κB pathway activation, activate Nrf2, downregulate TRPV1/TRPA1.	(75)
	carrageenan	IV (10 μg/kg)	Thermal pain↓	N/A	N/A	(57)
LXB4	carrageenan	IV (10 μg/kg)/IT (10 μg/kg)	Thermal pain↓	N/A	N/A	(57)

AITC, allyl isothiocyanate; CCD, chronic compression of the dorsal root ganglia; CFA, Complete Freund’s Adjuvant; CP, chronic pancreatitis; EAP, experimental autoimmune prostatitis; IP, intraperitoneal; IPL, Intraplantar; IT, intrathecal; IV, intravenous; LPS, lipopolysaccharide; LPV, localized provoked vulvodynia; MSU, monosodium urate; OA, osteoarthritis N/A, Not Available.

SPMs also alleviate nociception resulting from arthritis (**Table 3**). In mice with gouty arthritis, both intrathecal and intraperitoneal pretreatment with RvD1 (administered 72 hours before disease induction) alleviated mechanical hyperalgesia, with the 72-hour pretreatment window showing maximal efficacy. Dose-response studies revealed comparable effects at 3 ng and 30 ng RvD1, both superior to the 0.3 ng dose (54). A single dose of AT-RvD1 administered on day 3 post-induction alleviated mechanical hyperalgesia for 6 hours (42). Notably, MaR1 demonstrated delayed yet sustained efficacy: when administered every other day during peak joint inflammation (days 5–11 post-CFA), the first dose lacked acute anti-nociceptive effects. However, subsequent doses progressively reversed mechanical hypersensitivity, with analgesia persisting until day 25—well beyond the resolution of joint swelling and despite ongoing hyperalgesia (55).

SPMs play a role in carrageenan-induced nociception (**Table 3**). Both RvD1 and RvE1 were effective in preventing carrageenan-induced nociception; however, their combined use showed no additive or synergistic effects compared to monotherapy (41). Intrathecal pretreatment with LXA4 or 17(R)-RvD1 reduced the hyperalgesia index, with effects observed within the first 6 hours post-administration (56). Similar to LXA4, intravenous or intrathecal injection of ATLa (a more stable ATL analog) exerts comparable efficacy in alleviating carrageenan-induced hyperalgesia. However, 8,9-aLXB4 [(8,9)-acetylenic LXB4, an LXB4 analog] requires a higher dose to achieve anti-nociceptive effects similar to those of LXB4 (57).

Additionally, SPMs have been shown to exert effects in other models of nociception. RvD1 also exhibited prolonged efficacy in a trinitrobenzene sulfonic acid-induced visceral pain model, alleviating mechanical allodynia in a dose-dependent manner. Anti-nociceptive effects emerged 2 hours post-administration, peaked at 4 hours, and persisted for ≥12 hours before resolving by 24 hours (39). Both MaR1 and its precursor DHA markedly accelerate pain recovery in a mouse model of vulvodynia (58). RvD1, RvD2, PD1, MaR1, and MaR2 all inhibit capsaicin-evoked pain (37, 38, 53, 59, 60); RvD1, RvD2, RvE1, PD1, and MaR2 reduce formalin-evoked pain (36–38, 50, 53); and RvD2 and MaR2 attenuate AITC-evoked pain (38, 60).

### 2.3 Cancer pain

Pain is a prevalent and debilitating symptom among cancer patients, with approximately 40% experiencing inadequate pain relief despite current therapeutic regimens (61). Opioids remain the cornerstone of cancer pain management due to their potent anti-nociceptive efficacy. However, their use is frequently limited by adverse effects—including sedation, constipation, respiratory depression, and risk of dependence—that compromise both quality of life and, in some cases, overall survival (61). These challenges highlight the urgent need for novel analgesics that target pain mechanisms with greater specificity, thereby minimizing off-target effects while maintaining therapeutic efficacy.

SPMs exhibit divergent efficacy profiles in cancer pain models depending on the compound and disease context (**Table 4**). In the claw tumor model, RvD1 alleviated thermal hyperalgesia but had no significant effect on mechanical hypersensitivity. In contrast, RvD2 treatment reduced both mechanical and thermal hyperalgesia by day 21 post-tumor induction, though this effect declined by day 28 (62). Similarly, in a tongue tumor model, RvD1 failed to attenuate pain sensitivity, while RvD2 provided moderate analgesia; however, its effects were transient and lacked long-term sustainability (29). Notably, intrathecal RvD2 administration in a sarcoma model (NCTC cells injected into the distal femoral condyle) suppressed early-stage mechanical allodynia and thermal hyperalgesia. However, this anti-nociceptive effect was short-lived during later disease stages, suggesting time-dependent limitations in monotherapy (47). Additionally, while both RvD1 and RvE1 resolve bone cancer-induced mechanical pain comparably, RvD1 shows superior efficacy in alleviating thermal hyperalgesia at higher doses (63).

**Table 4:** Anti-nociceptive effect of SPM in cancer pain mode.

SPM molecules	Models	SPM administration routes	Effects	Relevant cells	Mechanisms	References
RvD1	Paw xenograft	IP (200 ng)	Thermal pain↓; No effect on mechanical pain	Immune cell /neuron /cancer cell	Inhibit TRP channels; reduce pro-inflammatory cytokines; modulate neurogenic inflammation.	(62)
	Bone cancer	IV (a single injection of 0.1 μg/kg or 1 μg/kg or daily injections of 10 μg/kg)	Mechanical pain↓	Spinal cord /Microglia /astrocyte	Inhibit central sensitization and release of inflammatory factors.	(155)
	Bone cancer	IT (0.03 or 1 ng)	Mechanical pain↓ Thermal pain↓	Spinal neuron /glial cell	Increase spinal levels of AEA and 2-AG; activate cannabinoid CB2 receptor.	(63)
RvD2	Paw xenograft/Tongue xenograft	IP (200 ng)	Paw cancer-induced thermal and mechanical pain↓ Tongue cancer-induced oral function↑	Cancer cell /Neutrophil /Macrophage	Inhibit IL-6/CXCL10; reduce necrosis and HIF-1α/CA9; decrease neutrophil infiltration and myeloperoxidase activity.	(62)
	Bone cancer	IT (500 ng)/IV (5 μg)	Mechanical pain↓ Thermal pain↓	Spinal cord /astrocyte	Inhibit the IL-17 CXCL1 neuroinflammatory axis in the spinal cord; attenuate astrocyte activation.	(47)
RvE1	Bone cancer	IT (0.03 or 1 ng)	Mechanical pain↓ Thermal pain↓	Primary afferent neuron /spinal neuron	Inhibit pain signaling by binding to and activating ChemR23 and BLT1 receptors	(63)

IP, intraperitoneal; IT, intrathecal; IV, intravenous.

### 2.4 Postoperative pain

Postoperative pain is a clinically significant challenge affecting the majority of patients following surgical procedures. Its intensity and duration are shaped by multifactorial determinants, including surgical invasiveness, patient-specific variables (e.g., age, sex, psychological state, comorbidities), and the adequacy of perioperative anti-nociceptive strategies (64). Despite advances in pain management, over 80% of patients report postoperative pain, with many experiencing inadequate relief due to suboptimal prevention or treatment protocols (64). This high prevalence underscores the urgent need for mechanism-driven, personalized approaches to improve both preoperative risk mitigation and postoperative pain control.

The action of SPMS in postoperative pain is summarized in **Table 5**. In the lateral paw incision model, prophylactic administration of RvD1 (20 ng, 30 minutes pre-surgery) produced sustained analgesia lasting 10 days postoperatively. Postoperative administration (20 ng or 40 ng on day 1) rapidly normalized pain thresholds to presurgical levels within 1 hour (65). In the skin/muscle incision and retraction model, early RvD1 treatment (day 2 post-surgery) prevented the development of skin/muscle incision and retraction-associated pain; delayed administration (day 9 or 17 post-surgery) provided only short-term relief, highlighting the critical window for therapeutic intervention (65). Similarly, in a thoracotomy model, RvD1 administered preoperatively or on postoperative day 4 attenuated pathological pain. Treatment initiated on or after day 14 showed no efficacy, further underscoring the importance of timing in SPM-based analgesia (66). In a postoperative pain mouse model induced by femoral fracture, MaR1 and RvD5 proved to be more effective than RvD1 and PD1 in providing pain relief, suggesting context-specific advantages (67). While DHA may transiently reduce perioperative pain through partial conversion to SPMs, its effects are markedly less potent than those of preformed SPMs, underscoring the limitations of relying on endogenous biosynthesis for pain therapy (67).

**Table 5:** Anti-nociceptive effect of SPM in postoperative pain models.

SPM molecules	Models	SPM administration routes	Effects	Relevant cells	Mechanisms	References
RvD1	LPI	IT (preoperative: 20 ng; postoperative: 20 or 40 ng)	Postoperative pain ↓	N/A	N/A	(65)
	SMIR	IT (early: 30 ng; later: 30 or 40 ng)	Postoperative pain ↓			(65)
	CPTP	IT (30 ng/30 μL)	Mechanical pain↓	N/A	N/A	(66)
RvD2	CPTP	IT (30 ng/30 μL)	Abnormal pain↓			(66)
RvD5	Bone fracture	IV (300 ng)	Mechanical pain in males↓	N/A	N/A	(136)
RvD5_n-3 DPA_	Bone fracture	IV (300 ng)	Mechanical pain in males↓			(136)
MaR2	Oral incision	IT (10 ng)	Thermal and mechanical pain↓	TG neuron	Reduce c-fos activation and the number of CGRP/p-NFκB-positive neurons in TG.	(50)

CPTP, chronic post-thoracotomy pain; IT, intrathecal; LPI, lateral paw incision; SMIR, skin/muscle incision and retraction. N/A, Not Available.

## 3 Mechanism of action of SPMs

### 3.1 Peripheral mechanism

**Figure 1:**
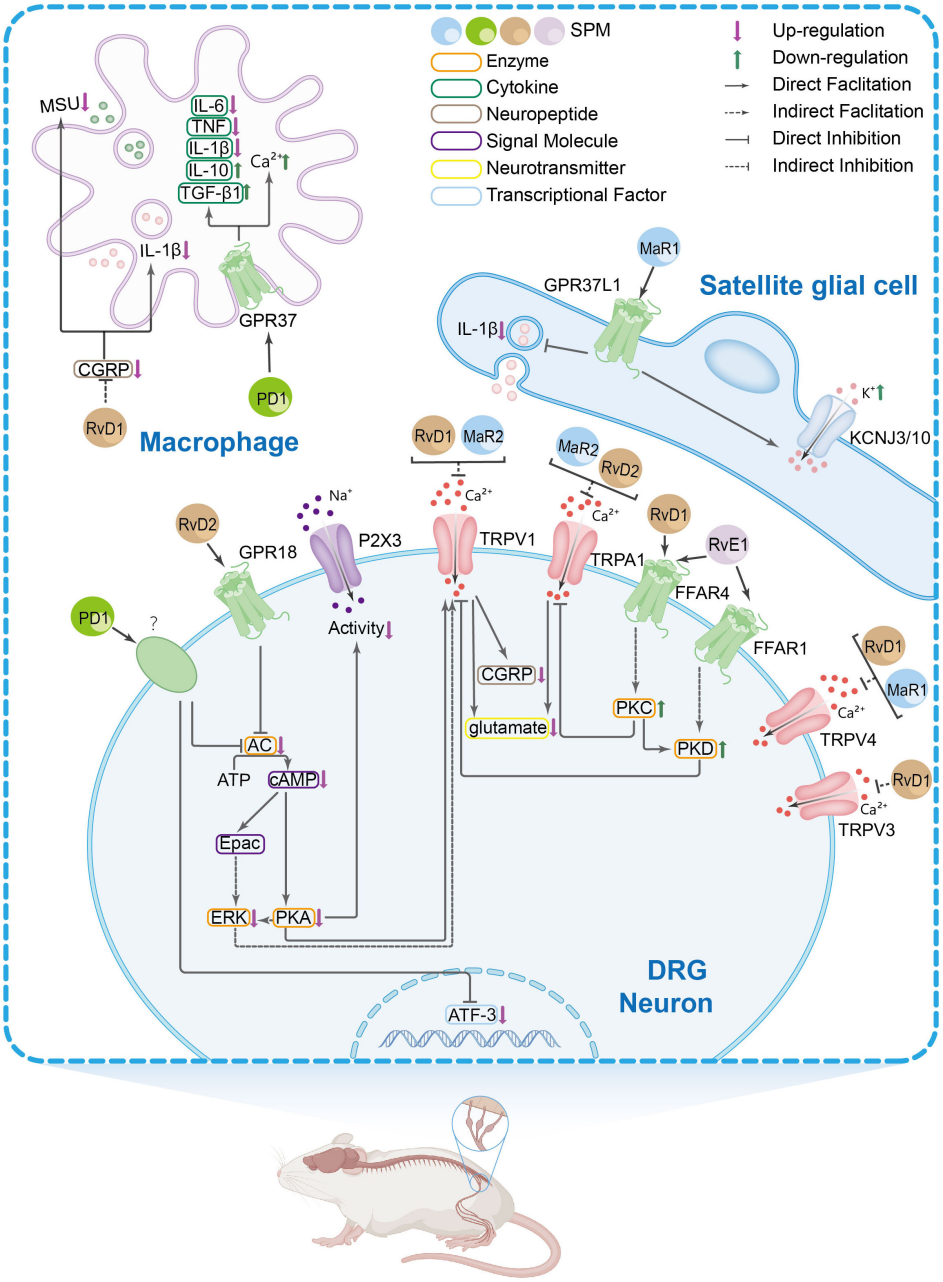
The main peripheral mechanisms of SPM’s anti-nociceptive effect in the dorsal root ganglia (DRGs) and macrophages. Mechanism of action of SPM at the DRG and macrophages level: 1. In DRG neurons: SPMs suppress transient receptor potential vanilloid subtype 1 (TRPV1) and TRPA1 activity, thereby reducing spinal dorsal horn release of CGRP and glutamate. This inhibition attenuates CGRP-induced interleukin-1β (IL-1β) release and monosodium urate (MSU) in macrophages. Evidence also suggests that SPMs may block TRPV1 signaling by suppressing AC-PKA-extracellular signal-regulated kinase (ERK) pathway activity and inhibiting the FFAR/protein kinase C (PKC) signaling axis and its effector PKD. The inhibition of TRPA1 by SPM is, rather, exerted by suppressing the free fatty acid receptor 4 (FFAR4)-PKC pathway. SPM targets GPR18 to inhibit the AC/cyclic adenosine monophosphate (cAMP)/PKA signaling pathway, thus inhibiting the P2X3 receptor. 2. In satellite glial cells: SPM acts on GPR37L1 to suppress IL-1β and promote KCNJ10-mediated potassium influx. 3. In macrophages: In the knee joint, RvD1 reduces macrophage phagocytosis of MSU crystals and the maturation and release of IL-1β. In natural macrophages, SPMs activate GPR37, elevating intracellular calcium concentrations to enhance phagocytic capacity. Concurrently, they inhibit the release of pro-inflammatory cytokines [e.g., IL-6, IL-1β, tumor necrosis factor alpha (TNF-α)] from peritoneal macrophages while promoting anti-inflammatory mediators such as IL-10 and transforming growth factor-β 1(TGF-β1).

**Figure 2:**
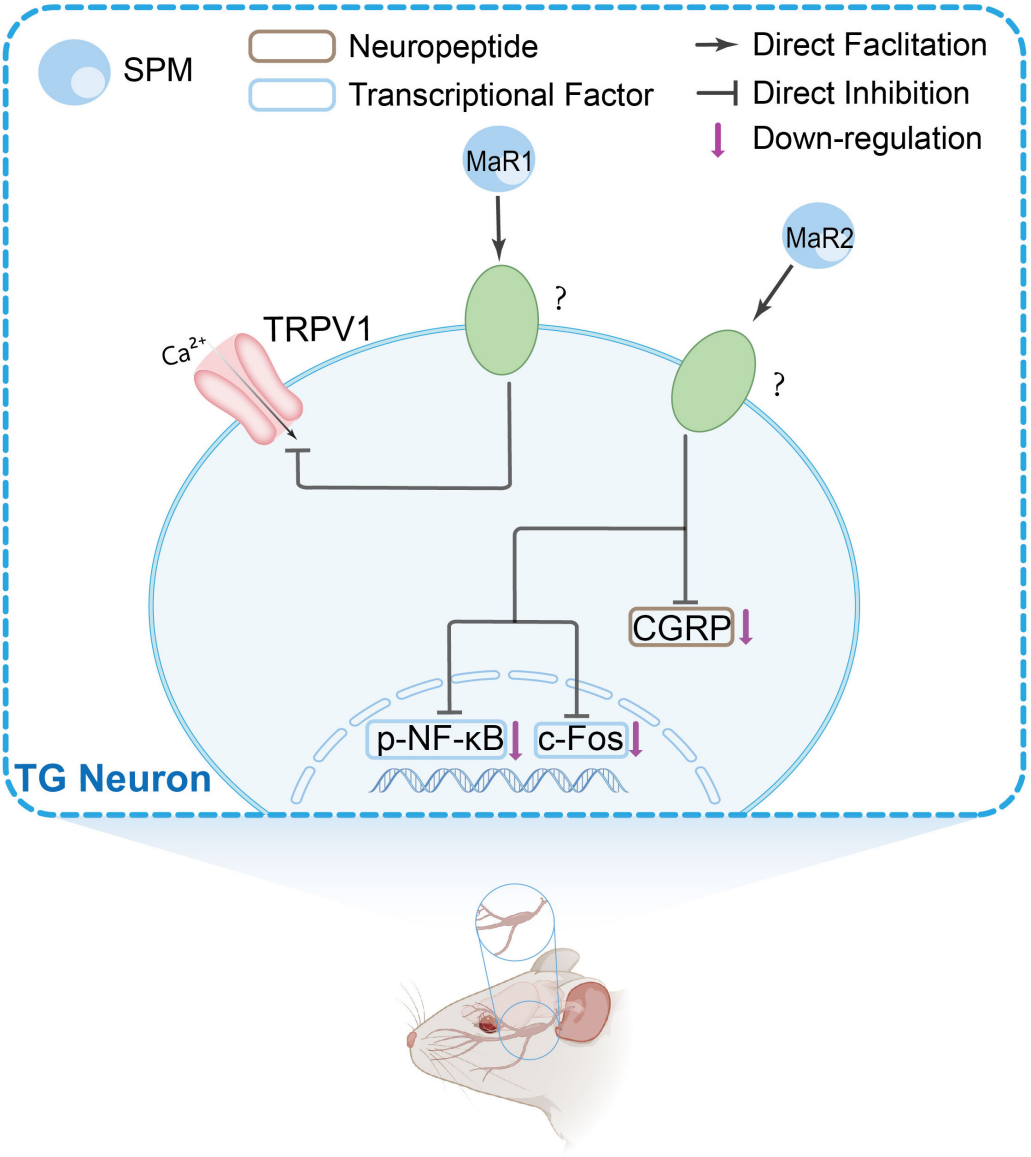
The main peripheral mechanisms of SPM’s anti-nociceptive effect in the trigeminal ganglia (TGs). Mechanism of action of SPMs in TG: SPMs inhibit TRPV1 channel activity, reduce CGRP levels, and decrease phosphorylated nuclear factor-κB (NF-κB) and c-fos expression, suggesting a role in modulating trigeminal nociceptive signaling.

Peripheral immune and glial cells contribute to pain signaling through neuro-immune crosstalk and inflammatory reactions, which play a role in pain sensitization and chronic pain (68–71). Research suggests that in the peripheral nervous system (PNS), SPMs show activity in the dorsal root ganglion (DRG) and trigeminal ganglion (TG). Current evidence indicates these effects may involve modulation of inflammatory cytokine signaling, TRP channels, G protein-coupled receptor (GPCR) activity, macrophage function, calcitonin gene-related peptide (CGRP) signaling, COX activity, and oxidative stress pathways. The mechanisms in DRG and TG are shown in **Figures 1** and **2**, respectively.

#### 3.1.1 Cell-specific effects in PNS

##### 3.1.1.1 Neuron-immune crosstalk in PNS

SPMs regulate pain by modulating immune cell function, particularly macrophage polarization (**Figure 1**). The classification of macrophage phenotypes traditionally follows the M1-M2 dichotomy (72), wherein M1 macrophages are pro-inflammatory, and M2 macrophages play anti-inflammatory roles and assist in tissue repair (73). At seven days post-spinal cord injury (SCI), cluster of differentiation 68 (CD68) (a marker for M1 macrophages) was up-regulated, while NT-3 (a marker associated with the M2 phenotype) was notably down-regulated. Intrathecal administration of RvD3 completely reversed this pattern, demonstrating that RvD3 promotes macrophage polarization toward the M2 phenotype (74). By favoring M2 polarization, these SPMs reduce pro-inflammatory cytokine release (e.g., TNF-α, IL-6) while amplifying anti-inflammatory mediators (e.g., IL-10, TGF-β). This rebalancing of macrophage activity not only mitigates inflammatory pain but also addresses the underlying tissue pathology driving nociceptive sensitization. LXA4 therapy reduces titanium dioxide-induced recruitment of total white blood cells, monocytes, and polymorphonuclear cells. In TiO_2_-driven inflammation, CD45^+^F4/80^+^ macrophages—the dominant monocyte-derived population—are the primary cellular target of LXA4. Specifically, LXA4 suppresses their recruitment and inhibits NF-κB activation within this macrophage subset, demonstrating a dual anti-inflammatory mechanism focused on this key cell type (75).

GPR37 exerts anti-inflammatory and anti-nociceptive effects primarily through its activity in macrophages. Upon activation by PD1, GPR37 signaling induces intracellular Ca2+ influx in macrophages, enhancing their phagocytic capacity. PD1 also promotes M2 macrophage differentiation via GPR37 activation, reducing pro-inflammatory cytokines (IL-1β, TNF, IL-6) and increasing anti-inflammatory ones (IL-10, TGF-β1). Preclinical studies indicate that PD1-GPR37 signaling augments macrophage-mediated pathogen clearance, which may contribute to its therapeutic potential in bacterial infections, sepsis, and malaria-associated complications. These mechanisms also correlate with alleviation of infection-related acute and chronic pain (29).

In the formalin-induced inflammatory pain model, RvE1’s anti-nociceptive effect is mediated by Gi-coupled GPCR ChemR23, which is expressed in DRG and spinal neurons (36). RvE1 directly activates ChemR23, and knockdown of ChemR23 abolishes RvE1’s anti-nociceptive action, linking ChemR23 to RvE1’s anti-inflammatory and anti-nociceptive effects (36). In human peripheral blood monocytes, RvE1 binds ChemR23 to activate the ERK/MAPK signaling pathway. In dendritic cells, RvE1 blocks IL-12 production (76).

##### 3.1.1.2 Neuron-glial crosstalk in PNS

In the peripheral nervous system, SPMs also exert their resolution action via glial cells. Through the MaR1/GPR37L1 signaling axis, PDX enhances surface expression and functional activity of KCNJ10 (Kir4.1) in satellite glial cells. Additionally, PDX inhibits PTX-induced interleukin-1β release in satellite glial cells-neuron co-cultures, suggesting dual mechanisms of action: potentiating Kir4.1-mediated homeostasis and suppressing neuroinflammatory signaling (77).

#### 3.1.2 Molecular mechanisms in PNS

##### 3.1.2.1 Inflammasome-, cytokine/chemokine- and Prostaglandin regulation in PNS

The NLR family pyrin domain containing 3 (NLRP3) inflammasome, a key driver of chronic pain pathogenesis, represents a promising therapeutic target for resolving persistent inflammation (78). SPMs modulate NLRP3 activity through distinct mechanisms. RvD1 inhibits NLRP3 inflammasome activation in mouse prostate tissue and alleviates pelvic pain and inflammation in preclinical models (5). In the spinal nerve ligation model, RvD1 alleviates mechanical allodynia by upregulating ALX/FPR2 receptor expression and concurrently inhibiting NLRP3 inflammasome assembly (79). RvD1 inhibits the phosphorylation of the ERK signaling pathway, consequently reducing the expression of NLRP3 inflammasome components and downstream cytokines (IL-1β and IL-18) mediated by phosphorylated ERK. This indicates anti-nociceptive action through the ERK/NLRP3/IL-1β inflammatory pathway (79). RvD2 promotes NLRP3 degradation via autophagy-dependent pathways, as evidenced by the reversal of its anti-IL-1β effects with autophagy inhibitors (bafilomycin A, 3-MA) but not proteasome inhibitors (MG-132) (80).

Notably, 17(R)-hydroxy-docosahexaenoic acid diminishes phosphorylation of the p65 subunit of NF-κB in the DRG three days post-adjuvant-induced arthritis induction (42). In the CCI-ION model, MaR2 reduces the number of TG neurons exhibiting phosphorylated NF-κB (50). Both RvD1 and LXA4 treatment inhibit the expression of NF-κB/p65 in the DRG (81, 82) (**Figure 2**).

SPMs modulate the production of inflammatory and anti-inflammatory cytokines/chemokines in pain states. RvD1 suppresses pro-inflammatory cytokines, including TNF-α, IL-18, and IL-1β, while enhancing anti-inflammatory mediators such as IL-10 and TGF-β1 (79, 82). AT-RvD1 reduces TNF-α and IL-1β levels in the ipsilateral hind paw, with greater efficacy in attenuating TNF-α (42). RvD2 alleviates cancer-induced hyperalgesia by suppressing IL-6 production in tumor and immune cells (62). LXA4 inhibits the overexpression of TNF-α, IL-1β, and IL-6 in the DRG and knee joint washes, and it can also enhance the production of IL-10 in knee joint washes (75, 81). MaR1 suppresses pro-inflammatory mediators, including TNF-α, inducible nitric oxide synthase (NOS2), and microRNA 155 (miR-155) (55). MaR2 downregulates pro-inflammatory cytokines (IL-1β, TNF-α, IL-6), chemokines [C-C motif chemokine ligand 2 (CCL2), CCL3, CCL17], and vascular endothelial growth factor by inhibiting neutrophil and monocyte infiltration into inflamed tissues, thereby disrupting immune-mediated hyperalgesia (60).

COX-2 is a key mediator of inflammatory pain, driving prostaglandin synthesis that sensitizes nociceptors and amplifies spinal nociceptive transmission (42). 17(R)-HDoHE, a docosahexaenoic acid-derived SPM, suppresses COX-2 overexpression in DRG neurons (42).

##### 3.1.2.2 Oxidative stress in PNS

RvD1 activates the nuclear factor erythroid 2-related factor 2/heme oxygenase-1 signaling axis, a critical regulator of cellular redox balance. By enhancing HO-1 expression, RvD1 reduces oxidative stress via scavenging of reactive oxygen species (ROS) while suppressing ROS-induced nociceptor sensitization and neuronal hyperexcitability (5). PDX treatment reduced ROS in serum (83). LXA4 restores antioxidant capacity through three interconnected mechanisms: it directly scavenges free radicals, including the model radical 2,2-azino-bis(3-ethylbenzothiazoline-6-sulfonate); upregulates the critical endogenous antioxidant glutathione by enhancing nuclear factor erythroid 2-related factor 2 (Nrf2) mRNA expression to drive antioxidant gene transcription; and reduces ROS levels, thereby mitigating oxidative stress and reestablishing redox homeostasis (75).

##### 3.1.2.3 Pain-related ion channels and neuropeptides in PNS

SPMs can inhibit the activation and upregulated expression of nociceptive TRP and purinergic P2X channels, which are key drivers of chronic pain (84, 85). Their modulation of ion channels and associated neuropeptides represents a significant mechanism for resolving pain.

Among resolvins, RvD1 exerts broad analgesic effects by inhibiting TRPA1, TRPV3, and TRPV4 at nanomolar to micromolar concentrations. Its peripheral administration attenuates agonist-evoked acute pain, while pretreatment reverses inflammatory mechanical and thermal hypersensitivity (53). RvD1 appears to inhibit TRPA1 channel activity via the FFAR4-PKC signaling axis (86). A metabolically stable RvD1 analog, 17R-RvD1, selectively inhibits TRPV3 channels, highlighting its potential for pain related to TRPV3 hyperactivity (87). In contrast, RvD2 indirectly suppresses TRPV1 and TRPA1 currents (38). Similarly, RvD3’s inhibition of TRPV1 in DRG neurons is mediated through the ALX/FPR2 receptor (21). RvE1 exhibits a multimodal action, alleviating both TRPV1-dependent and independent pain. Its effects are dose-dependent (at low concentrations it suppresses TRPV1 activity, while at high concentrations it further modulates TRPA1), and involve multiple receptors (FFAR1/FFAR4) and pathways (PKD/PKC) (36, 86). In cultured small-diameter DRG neurons, RvE1 also inhibits substance P-mediated TRPV1 activity (88). Beyond resolvins, other SPMs also inhibit TRP channels. PD1 and MaR1 specifically inhibit TRPV1 via Gαi-coupled GPCRs, without affecting TRPA1, highlighting their selectivity (37, 55, 59, 89). MaR1 also rapidly terminates TRPV4 signaling, broadening its antinociceptive scope (90). In contrast, MaR2 and LXA4 exhibit a broader inhibitory profile, suppressing both TRPV1 and TRPA1 (60). In addition, RvD2 also mediates analgesia by activating GPR18, which couples to pertussis toxin-sensitive Gαi/o proteins. RvD2/GPR18 signaling cascade further inhibits ionotropic P2X3 receptor activity by suppressing the cAMP/PKA pathway (91).

SPMs also alleviate pain by modulating key neuropeptides like CGRP. MaR1 suppresses neutrophil/macrophage recruitment near CGRP+ fibers in the hind paw skin and reduces CGRP release from DRG neurons (92). MaR2 concentration-dependently reduces CGRP release evoked by TRPV1/TRPA1 agonists from sensory neurons, and attenuates TRPV1/TRPA1-mediated nociception (60). Similarly, MaR2 reverses activation of CGRP+ neurons in TG in a model of trigeminal neuropathic pain (CCI-ION) (50).

### 3.2 Central mechanism

**Figure 3:**
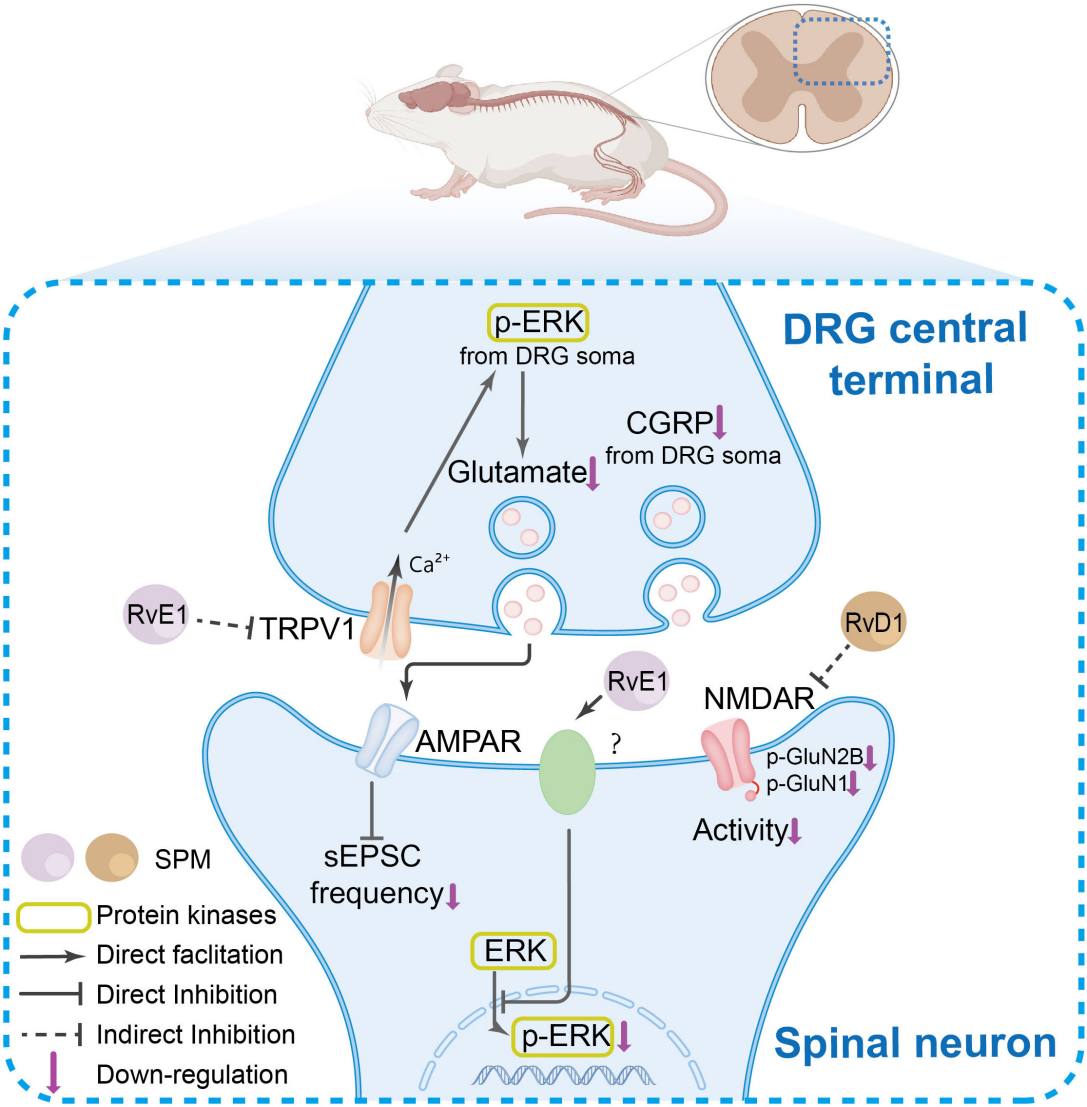
The SPM’s anti-nociceptive mechanisms in spinal neurons and synaptic transmission. In the DRG central terminals, under pain conditions, TRPV1 channel activation and ERK phosphorylation, which collectively enhance spinal synaptic plasticity and central sensitization. SPMs suppress TRPV1 activation, thereby inhibiting ERK phosphorylation in DRG central terminals. This reduction in phosphorylated ERK diminishes its facilitatory effect on glutamate release from presynaptic terminals in the spinal cord, ultimately attenuating α-Amino-3-hydroxy-5-methyl-4-isoxazolepropionic acid receptor (AMPAR)-mediated synaptic plasticity in spinal neurons. Furthermore, SPMs inhibit N-methyl-D-aspartate receptor (NMDAR) hyperactivity and ERK phosphorylation, modulating nociceptive signal transmission.

**Figure 4:**
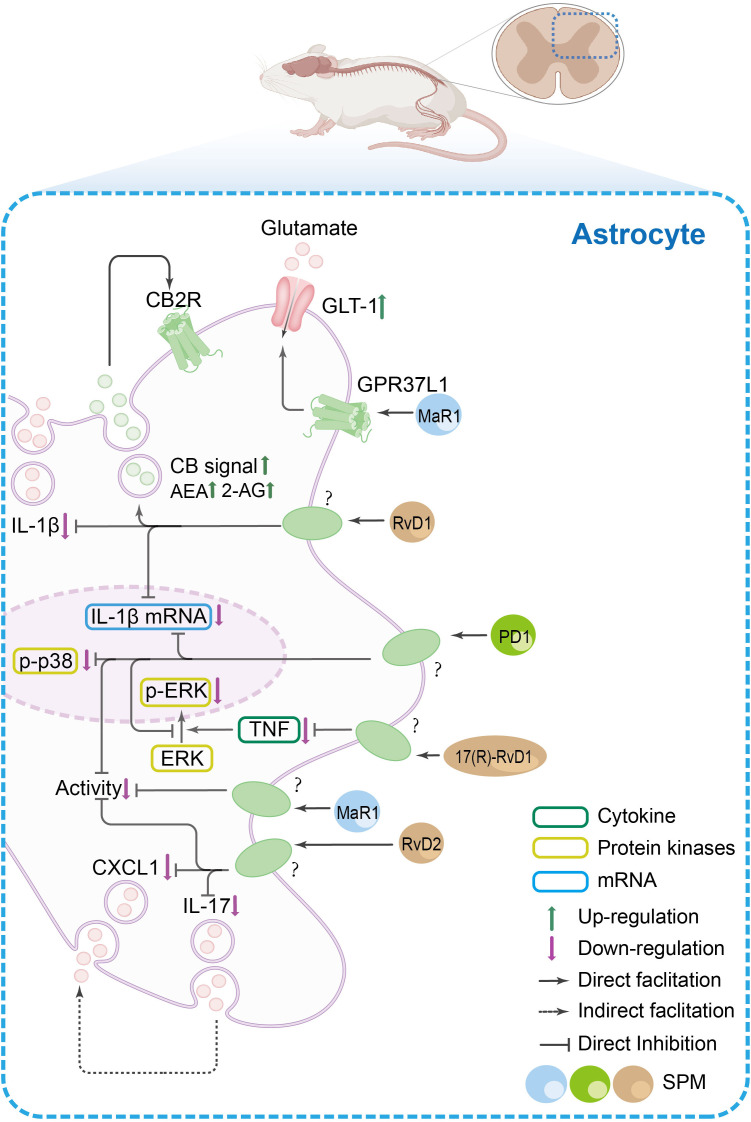
The SPM’s anti-nociceptive mechanisms in spinal astrocytes. In spinal astrocytes, inflammatory pain states are characterized by increased release of pro-inflammatory cytokines (e.g., IL-1β, IL-17), chemokines [e.g., C-X-C motif chemokine ligand 1(CXCL1)], and glutamate, alongside reduced endocannabinoid levels and elevated p38 mitogen-activated protein kinase/ERK phosphorylation. SPMs exert dual regulatory effects: (1) they promote the release of anti-inflammatory mediators such as endocannabinoids, which act on cannabinoid 2 receptor (CB2R) in glial cells to amplify anti-nociceptive signaling, and (2) they suppress pro-inflammatory mechanisms by inhibiting IL-1β production, p38/ERK phosphorylation, and CXCL1/IL-17 release. Additionally, SPMs enhance glutamate clearance via GPR37L1-mediated activation of glutamate transporter 1 (GLT-1).

**Figure 5:**
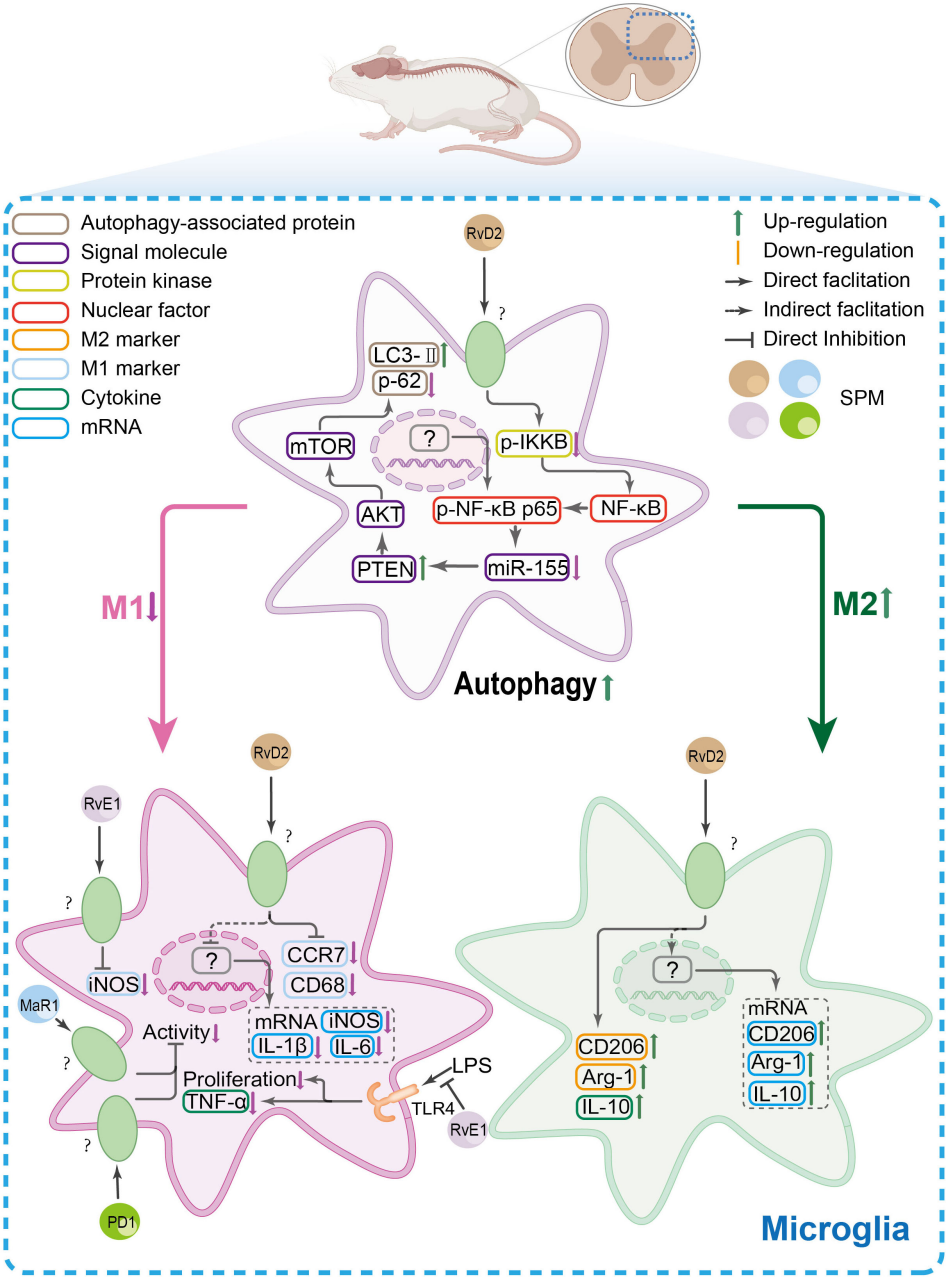
The SPM’s anti-nociceptive mechanisms in spinal microglia. In spinal microglia, inflammatory conditions shift their polarization toward a pro-inflammatory M1 phenotype [marked by elevated CC-chemokine receptor 7(CCR7), CD68, and inducible nitric oxide synthase (iNOS)] while suppressing the anti-inflammatory M2 phenotype [CD206, arginase-1(Arg-1), and IL-10]. Concomitantly, autophagy is downregulated. SPMs reverse this imbalance by promoting M2 polarization, suppressing TNF-α-driven microglial inflammation, and restoring autophagy via the NF-κB/miR-155 axis, which targets the phosphatase and tensin homolog (PTEN)/protein kinase B/mammalian target of rapamycin (mTOR) pathway.

The spinal cord serves as a critical hub for nociceptive signal transmission and processing. Current research suggests that SPMs exert antinociceptive effects in the central nervous system through several potential mechanisms, including: modulation of spinal synaptic plasticity, regulation of inflammatory cytokine signaling, interaction with glial cells, influence on GPCR-mediated pathways, alteration of CGRP signaling, effects on autophagy processes, and modulation of COX and oxidative stress pathways. These proposed mechanisms are summarized in **Figure 3**, **Figure 4**, and **Figure 5**.

#### 3.2.1 Cell-specific effects in the spinal cord

##### 3.2.1.1 Neuron-glial crosstalk in the spinal cord

Spinal astrocytes and microglia are central regulators of neuroinflammation and pain pathogenesis (93–95). In astrocytes, which contribute to pain maintenance through metabolic coupling with neurons (96), RvD2 exerts its effect by inhibiting interleukin-17 secretion and the subsequent release of the chemokine CXCL1, thereby attenuating astrocyte-mediated neuroinflammation (47). In microglia, key amplifiers of nociception via synaptic remodeling and cytokine production (97), various SPMs demonstrate inhibitory effects. AT-RvD1 reduces SNL-induced expression of the microglial marker Iba1 (98). In primary microglial cultures, RvE1 alleviates neuropathic pain by inhibiting lipopolysaccharide (LPS)-induced proliferation and the synthesis/release of TNF-α (48). RvD1 suppresses microglial activation and reduces phosphorylated p38 levels; it further exerts anti-inflammatory effects by modulating the brain-derived neurotrophic factor (BDNF)/tropomyosin-related kinase B (TrkB) signaling pathway, which participates in a positive feedback loop with p38 to amplify pro-inflammatory responses (99, 100). Lipoxin A4 (LXA4), while not affecting spinal cord injury (SCI)-induced astrocytosis, specifically reduces microglial reactivity and suppresses interferon-gamma-induced TNF-α release (101).

Notably, several SPMs exhibit broad-spectrum inhibition across glial cell types. For instance, pretreatment with protectin D1 (PD1) inhibits the robust microglial and astrocytic activation triggered by nerve injury (40). Similarly, MaR1 reduces the activation of both cell types in the spared nerve injury (SNI) model, as indicated by decreased expression of Iba1 and glial fibrillary acidic protein (GFAP), an effect that is sex-independent (102).

#### 3.2.2 Molecular mechanisms in the brain and spinal cord

##### 3.2.2.1 Inflammasome-, cytokine/chemokine- and prostaglandin regulation in the spinal cord

Within the spinal cord, SPMs orchestrate the resolution of inflammation by targeting key signaling hubs, such as the NLRP3 inflammasome, the NF-κB and MAPK pathways, and the downstream cytokine and COX networks, in order to alleviate pain.

In the spinal cord, RvD1 inhibits the SNL-induced upregulation of the NLRP3 inflammasome complex and associated nociceptive behavior by suppressing the ERK/NLRP3/IL-1β signaling axis (79). Similarly, MaR1 inhibits NLRP3 inflammasome activation and pyroptosis in inflammatory pain models (103, 104). SPMs also target MAPK and NF-κB pathways, which regulate inflammasome assembly and pro-inflammatory gene transcription. LXA4 attenuates phosphorylated ERK expression in a dose-dependent manner (105), while PD1 inhibits the phosphorylation of both p38 and ERK. Concurrently, multiple SPMs, including MaR1, 17(R)-HDHA, RvD1, and LXA4, suppress the activation or phosphorylation of NF-κB in various pain models (38, 82, 92, 105). RvD2 further enhances autophagic flux through an NF-κB-regulated miR-155/PTEN axis, promoting a microglial phenotypic switch to the anti-inflammatory M2 state (106).

By targeting these signaling hubs, SPMs comprehensively reprogram the spinal cytokine synthesis and release. They effectively suppress a broad spectrum of pro-inflammatory mediators (e.g., IL-1β, IL-6, IL-18, TNF-α, CCL2) while promoting the production of anti-inflammatory cytokines (e.g., IL-10, TGF-β). RvD1, AT-RvD1, and RvD5 suppress IL-6, IL-18, TNF-α, and IL-1β, while enhancing IL-10 and TGF-β1 (39, 79, 82, 98, 107). RvD2 specifically inhibits IL-17 secretion (47). PD1/PDX downregulates TNF-α, IL-1β, and CCL2, while upregulating TGF-β. PDX reverses non-compressive lumbar disc herniation (NCLDH)-induced increases in IL-6/IL-1β and decreases in TGF-β mRNA (40, 108, 109). MaR1 inhibits the production of multiple chemokines and cytokines (e.g., CXC12, CXCL1, CCL3/4, IL-1β, IL-6, IL-18, TNF-α), likely via STAT and MAPK pathways (92, 103, 110, 111). LXA4 and its analog ATL inhibit TNF-α and IL-1β while upregulating TGF-β1 and IL-10, with ATL also suppressing IL-1β, IL-6, and TNF-α mRNA levels (105, 112).

Beyond cytokine networks, SPMs also target enzymes critical for prostaglandin synthesis. For instance, 17(R)-HDHA downregulates spinal COX-2 mRNA, thereby interrupting prostaglandin-driven hyperalgesia (42).

##### 3.2.2.2 Pain-related channels and neuropeptides in the spinal cord

CB2 receptors are predominantly expressed on neuroglial cells. RvD1 has been shown to elevate spinal levels of the endocannabinoids anandamide (AEA) and 2-arachidonoylglycerol (2-AG). Given that the receptors for RvD1 are also primarily localized on spinal glial cells, these cells are likely the principal sites through which RvD1 induces the upregulation of AEA and 2-AG. Subsequently, these endocannabinoids activate CB2 receptors, leading to the inhibition of pro-inflammatory signaling and reduced sensitization of nociceptors (63).

SPMs modulate pain by targeting the interplay between CGRP and sirtuin 1 (SIRT1), key regulators of neuroinflammation and nociception. In the NCLDH model, PD1 downregulates CGRP (a potent pro-nociceptive neuropeptide) while upregulating SIRT1 (a deacetylase with anti-inflammatory and neuroprotective roles) in the NCLDH model (108). CGRP exacerbates pain by enhancing TNF-α/IL-1β (pro-inflammatory) and suppressing TGF-β (anti-inflammatory). Conversely, SIRT1 counteracts this by reducing TNF-α/IL-1β and elevating TGF-β (108). Additionally, intrathecal injection of EX-527 (a classic SIRT1 enzyme activity inhibitor) results in increased activation of CGRP, while administration of SIRT1720 (a specific agonist of SIRT1) reduces CGRP expression in the spinal cord (108). Furthermore, administration of CGRP and a CGRP antagonist influences SIRT1 release, with CGRP reducing and the antagonist promoting SIRT1 release (108). This reciprocal inhibition highlights a CGRP-SIRT1 axis central to NCLDH pain (108). These findings indicate an interaction between CGRP and SIRT1, suggesting that PD1 alleviates pain by restoring SIRT1-mediated suppression of CGRP signaling, rebalancing inflammatory and reparative pathways (108). These findings establish SPMs as master regulators of the CGRP-SIRT1 axis, providing dual therapeutic benefits: anti-inflammatory effects by suppressing pro-nociceptive cytokines (such as TNF-α and IL-1β), and pro-resolution effects by enhancing anti-inflammatory mediators (like TGF-β) and the neuroprotective SIRT1.

##### 3.2.2.3 Oxidative stress in the brain

In a rat model of diabetic pain, PDX treatment reduced lipid peroxidation in pain-related brain regions, including the prefrontal cortex and hippocampus, with the hippocampus showing greater sensitivity to this effect. Furthermore, PDX potently suppressed ROS levels specifically in the hippocampus (83).

##### 3.2.2.4 Spinal synaptic plasticity/neurotransmitter regulation

A hallmark of chronic pain is central sensitization, characterized by enhanced spinal synaptic plasticity [e.g., long-term potentiation (LTP)] and neuronal hyperexcitability (38, 113). SPMs counteract this process through regulation of key ion channels and receptors essential in synaptic plasticity and neurotransmitter (e.g., glutamate) signaling.

SPMs effectively normalize maladaptive spinal synaptic plasticity. RvD2 inhibits inflammation-induced spinal synaptic plasticity by suppressing TRPV1/TRPA1 signaling. A comparative study showed that RvD1, RvE1, and RvD2 all attenuate TRPV1/TRPA1-mediated plasticity in a dose-dependent manner, with efficacy following RvD1 > RvE1 > RvD2 (38). Particularly, PD1 not only reverses neuropathy-induced synaptic plasticity but also prevents the induction of spinal LTP. PD1 also reduces TNF-α- and TRPV1-driven increases in spontaneous excitatory postsynaptic current (sEPSC) frequency without affecting baseline transmission (37). Notably, PD1 is more effective than equimolar doses of RvE1 in blocking LTP development (37, 40).

Beyond synaptic plasticity, SPMs modulate critical postsynaptic receptors. The NMDA receptor, particularly those containing the NR2B subunit, is pivotal for central sensitization. RvD1 suppresses NMDA receptor signaling by inhibiting the phosphorylation of NR1 and NR2B subunits in spinal dorsal horn neurons, thereby reducing receptor excitability (39). This NR2B phosphorylation can propagate pain via spinal astrocytes by activating c-Jun N-terminal kinase (JNK) and driving pro-inflammatory cytokine production (114). RvE1 may modulate nociceptive processing presynaptically by inhibiting TRPV1/TNF-α signaling and postsynaptically by suppressing ERK-dependent NMDA receptor activation, contributing to attenuated central sensitization (36).

SPMs dampen the pain-evoked neuron hyperexcitability. AT-RvD1, acting through the ALX/FPR2 receptor, reduces the hyperactivity of spinal wide-dynamic-range (WDR) neurons evoked by peripheral inflammation. Crucially, it selectively dampens pathological pain transmission while preserving baseline nociceptive function (20).

To note, SPMs can simultaneously regulate synaptic transmission through presynaptic, postsynaptic, and astrocyte-mediated mechanisms. MaR1 reverses TRPV1-mediated presynaptic hyperactivity (reflected in sEPSC frequency) and normalizes postsynaptic receptor sensitization (reflected in sEPSC amplitude), offering a comprehensive strategy to restore synaptic homeostasis (89). MaR1 also activates astrocytic GPR37L1, promoting its association with the glutamate transporter GLT-1. This enhances glutamate reuptake, reduces synaptic glutamate levels, and mitigates neuropathic pain in rodent models (28).

##### 3.2.2.5 Autophagy in the spinal cord

Autophagy, a crucial process for maintaining cellular homeostasis, plays a stage-dependent role in neuropathic pain. Its transient inhibition may initially delay neuroinflammation, but chronic impairment of autophagic flux sustains inflammatory responses and exacerbates pain (115). The AMP-activated protein kinase (AMPK) pathway serves as a key regulator linking cellular energy status to autophagic activity (116). AT-RvD1 promotes autophagy, as indicated by an increased LC3B-II/I ratio and accumulation of autophagy-related proteins, thereby attenuating NLRP3 inflammasome activation (98). In an NCLDH pain model, where pain is associated with suppressed AMPK signaling and impaired autophagy, the SPM analog PDX reverses this deficit. It restores phosphorylated AMPK/AMPK activity, normalizes autophagic flux, and ultimately alleviates neuropathic pain (109).

## 4 Summary and future directions

### 4.1 Therapeutic advantages of SPMs over conventional analgesics

SPMs offer a targeted and physiological approach to pain resolution by engaging natural resolution pathways, which distinguishes them from conventional analgesics. A key advantage is their ability to promote active inflammation resolution rather than merely suppressing inflammatory signals, leading to more complete tissue recovery and potentially improved long-term outcomes. Unlike non-steroidal anti-inflammatory drugs that globally inhibit inflammation, SPMs function in a context-dependent manner, resolving inflammation without compromising host defense mechanisms. Their multi-modal mechanism of action—simultaneously targeting ion channels on neurons, modulating glial cell activity, and reprogramming immune responses—allows for synergistic effects that address both peripheral and central sensitization processes. This comprehensive approach may explain their efficacy in diverse pain conditions while demonstrating a favorable safety profile in preclinical models. Notably, SPMs exhibit selective action on pathological pain states while preserving physiological nociceptive function, suggesting a reduced risk of side effects compared to broad-spectrum analgesics. Their ability to resolve neuroinflammation and reverse maladaptive plasticity further positions them as promising candidates for preventing the transition from acute to chronic pain. These inherent advantages, combined with emerging strategies to enhance their stability and bioavailability, underscore the potential of SPMs as a novel class of therapeutics that could complement or reduce reliance on current analgesics, particularly opioids.

### 4.2 Current gaps and mechanistic limitations

The current understanding of SPM mechanisms in pain resolution remains incomplete, constrained by several key limitations. Most research has focused on a narrow subset of SPMs—such as RvD1 and PD1—while the bioactivities and therapeutic potential of other members remain underexplored. This restricted focus not only limits a systems-level view of the resolvome but also leads to a related methodological gap: the general lack of head-to-head comparisons between different SPM subtypes within the same study. Without such direct comparative controls, it remains challenging to establish a clear hierarchy of efficacy or delineate unique versus shared functions among SPMs, thereby limiting the interpretation of their therapeutic potential. Furthermore, although SPMs are known to modulate immune cells and promote inflammatory resolution, their direct molecular targets and receptor-specific mechanisms are not fully elucidated. Receptors such as BLT1, ALX/FPR2, GPR32, ChemR23/CMKLR1, GPR18, GPR37, and LRG6 have been identified (see graphic abstract), yet a comprehensive mapping of SPM-receptor interactions across relevant cell types in pain pathways is still lacking (26). Moreover, the majority of mechanistic insights derive from macrophages and microglia, with far less attention paid to other types of cells. The roles of SPMs in regulating synaptic plasticity, ion channel activity, and neurotransmitter clearance within neural circuits therefore, require deeper investigation. Critically, the feedback effect of pain itself on SPM metabolism remains poorly understood. Chronic pain conditions may alter the expression of SPM biosynthetic enzymes or enhance degradation pathways, yet such adaptive mechanisms have not been systematically studied. The dynamic changes in endogenous SPM levels throughout different phases of pain progression also remain poorly characterized. For instance, one clinical study observed that migraine patients had significantly lower LXA4 levels than healthy controls, both during and between attacks, and those with attacks lasting longer than 12 hours showed even lower LXA4 levels than those with shorter attacks, suggesting that LXA4 depletion may accompany pain persistence (117). This clinical observation underscores the dynamic nature of the SPM system *in vivo*, further highlighting the importance of study designs that include appropriate controls—such as precursor molecules (e.g., DHA/EPA) or different SPMs—to distinguish specific pharmacological effects from broader, condition-dependent alterations in resolution pathways.

### 4.3 Sexual dimorphism and consideration for individualized therapy

Emerging evidence highlights sexual dimorphism in the anti-nociceptive effects of SPMs, with males and females exhibiting divergent responses across preclinical models. Specifically, intrathecal administration of RvD5 attenuates paclitaxel-induced mechanical allodynia (25), and it selectively inhibits the inflammatory phase of formalin-induced spontaneous pain in males (118). However, RvD5 shows no significant effect in female mice in either of these pain models. In male trigeminal neuralgia models, RvD5 downregulates IL-6 production specifically in male models of trigeminal neuralgia (118). Post-MaR1 treatment in the spared nerve injury model, males exhibit elevated macrophage colony-stimulating factor levels compared to females (102). The implications of sexual dimorphism in pain modulation highlight the importance of considering sex-specific mechanisms and therapeutic strategies. Specifically, sex hormones (e.g., testosterone, estrogen) likely interact to modulate the efficacy of SPMs, although the exact mechanisms remain unclear. These findings underscore the necessity for sex-balanced preclinical studies and tailored therapeutic approaches to optimize SPM-based pain management.

### 4.4 Challenges in clinical translation

The translation of SPMs from preclinical promise to clinical application remains at an early stage. Human studies have predominantly used SPM precursors (e.g., DHA, EPA) or intermediates (e.g., 17-HDHA and 18-HEPE) rather than pre-formed SPMs due to the latter’s poor bioavailability and metabolic instability (119–121). The reason may relate to the inherent instability of SPMs, which are sensitive to oxygen, light, and heat, raising concerns about shelf-life. However, precursor efficacy is inherently limited because PUFAs require enzymatic conversion to active SPMs via rate-limiting steps involving lipoxygenases and cyclooxygenases (26), explaining their reduced potency (43). Even excess PUFAs show limited therapeutic impact due to this metabolic bottleneck. This limitation is compounded in disease states where PUFA metabolism is impaired. Obesity disrupts 17-HDHA and PD1 biosynthesis (122); S. aureus infection in macrophages elevates COX-2/mPGES-1 but suppresses 15-LOX-1, impairing SPM production (123); and fatty acid desaturase 1 deficiency alters hepatic PUFA metabolism, limiting SPM synthesis (124). Thus, simply increasing precursor supply is ineffective when biosynthetic pathways are compromised, underscoring the rationale for using pre-formed SPMs. Clinical progress is further hindered by sexual dimorphism in SPM responses (26), heterogeneous trial designs, small cohorts, and varied control groups (120, 121, 125, 126). No large-scale trials have directly assessed fully synthesized SPMs, leaving a major translational gap.

### 4.5 Future research directions

Future strategies include both enhancing endogenous SPM production and developing exogenous supplementation. Elevating endogenous SPM biosynthesis through interventions is a promising strategy. For example, by increasing the activity of key enzymes such as lipoxygenases to improve precursor conversion efficiency. Several interventions—including high-intensity swimming (127), spinal cord stimulation (128), vagus nerve stimulation (129), the caspase-1 inhibitor VX-765 (104), soluble epoxide hydrolase (sEH) inhibition (130), and specific inhibitors such as SAFit2 (131) —have shown promise in elevating SPM levels in preclinical models. Given that particular pathological conditions like obesity may impair SPM production (122–124), exogenous supplementation with stabilized SPM analogs represents a more direct approach in several circumstances. Critical translational barriers include inherent SPM instability, necessitating advanced formulations (e.g., nano-encapsulation, structural modifications, prodrug designs) to improve shelf-life, half-life, and bioavailability (26, 132). Scalable synthetic production and rigorous safety profiling are essential. Developing non-invasive delivery routes (oral, transdermal) is crucial for clinical feasibility beyond intrathecal administration. Finally, exploring SPMs in combination therapy with conventional analgesics may yield synergistic effects, supporting a multi-mechanistic approach to pain management. Future clinical efforts must incorporate sex as a biological variable to ensure therapies are effective for all patients.

## Glossary

2-AG: 2-Arachidonoylglycerol
AEA: anandamide
ALX: lipoxin A receptor
AMPAR: α-Amino-3-hydroxy-5-methyl-4-isoxazolepropionic acid receptor
AMPK: AMP-activated protein kinase
Arg-1: arginase-1
ATF-3: activating transcription factor 3
cAMP: cyclic adenosine monophosphate
CB2R: cannabinoid 2 receptor
CCI: chronic constriction injury
CCL2: C-C motif chemokine ligand 2
CCR7: C-C chemokine receptor 7
CD68: cluster of differentiation 68
CFA: Complete Freund’s Adjuvant
CGRP: calcitonin gene-related peptide
ChemR23: chemerin receptor 23
COX-2: cyclooxygenase-2
CXCL1: C-X-C motif chemokine ligand 1
DHA: docosahexaenoic
DRG: dorsal root ganglion
DRV1: Resolvin D1 receptor 1
EPA: eicosapentaenoic
Epac: exchange protein activated by cAMP
ERK: extracellular signal-regulated kinase
FFAR4: free fatty acid receptor 4
FPR2: formyl-peptide receptor 2
GLT-1: glutamate transporter 1
GPCR: G protein-coupled receptor
GPR32: G protein-coupled receptor 32
IKKβ: IκB kinase β
IL-1β: interleukin-1β
iNOS: inducible nitric oxide synthase
LC3-II: microtubule-associated protein 1 light chain 3 II
LTP: long-term potentiation
miR-155: microRNA 155
MSU: monosodium urate
mTOR: mammalian target of rapamycin
NCLDH: non-compressive lumbar disc herniation
NF-κB: nuclear factor-κB
NLRP3: NLR family pyrin domain containing 3
NMDAR: N-methyl-D-aspartate receptor
p-62: sequestosome 1
PKC: protein kinase C
PNS: peripheral nervous system
PTEN: phosphatase and tensin homolog
sEPSC: spontaneous excitatory postsynaptic current
SIRT1: sirtuin 1
SPMs: specialized pro-resolving mediators
TG: trigeminal ganglion
TGF-β1: transforming growth factor-β
TNF-α: tumor necrosis factor alpha
TRPA1: transient receptor potential ankyrin subtype 1
TRPV1: transient receptor potential vanilloid subtype 1.
